# Fe-N co-doped carbon nanofibers with Fe_3_C decoration for water activation induced oxygen reduction reaction

**DOI:** 10.1093/nsr/nwae193

**Published:** 2024-06-04

**Authors:** Shaoxiong Li, Gengyu Xing, Sheng Zhao, Jian Peng, Lingfei Zhao, Feng Hu, Linlin Li, Jiazhao Wang, Seeram Ramakrishna, Shengjie Peng

**Affiliations:** College of Materials Science and Technology, Nanjing University of Aeronautics and Astronautics, Nanjing 210016, China; College of Materials Science and Technology, Nanjing University of Aeronautics and Astronautics, Nanjing 210016, China; College of Materials Science and Technology, Nanjing University of Aeronautics and Astronautics, Nanjing 210016, China; Institute for Superconducting and Electronic Materials Australian Institute for Innovative Materials, University of Wollongong Innovation Campus, North Wollongong, NSW 2522, Australia; Institute for Superconducting and Electronic Materials Australian Institute for Innovative Materials, University of Wollongong Innovation Campus, North Wollongong, NSW 2522, Australia; College of Materials Science and Technology, Nanjing University of Aeronautics and Astronautics, Nanjing 210016, China; College of Materials Science and Technology, Nanjing University of Aeronautics and Astronautics, Nanjing 210016, China; Institute for Superconducting and Electronic Materials Australian Institute for Innovative Materials, University of Wollongong Innovation Campus, North Wollongong, NSW 2522, Australia; Department of Mechanical Engineering, National University of Singapore, Singapore 117583, Singapore; College of Materials Science and Technology, Nanjing University of Aeronautics and Astronautics, Nanjing 210016, China

**Keywords:** electrospinning, nanofibers, Fe–N–C, oxygen reduction reaction, Al-air batteries

## Abstract

Proton activity at the electrified interface is central to the kinetics of proton-coupled electron transfer (PCET) reactions in electrocatalytic oxygen reduction reaction (ORR). Here, we construct an efficient Fe_3_C water activation site in Fe-N co-doped carbon nanofibers (Fe_3_C-Fe_1_/CNT) using an electrospinning-pyrolysis-etching strategy to improve interfacial hydrogen bonding interactions with oxygen intermediates during ORR. *In situ* Fourier transform infrared spectroscopy and density functional theory studies identified delocalized electrons as key to water activation kinetics. Specifically, the strong electronic perturbation of the Fe–N_4_ sites by Fe_3_C disrupts the symmetric electron density distribution, allowing more free electrons to activate the dissociation of interfacial water, thereby promoting hydrogen bond formation. This process ultimately controls the PCET kinetics for enhanced ORR. The Fe_3_C-Fe_1_/CNT catalyst demonstrates a half-wave potential of 0.83 V in acidic media and 0.91 V in alkaline media, along with strong performance in H_2_-O_2_ fuel cells and Al-air batteries.

## Teaser

Delocalized electrons driving dissociation of interfacial water promotes hydrogen bonding, thus improving the oxygen reduction kinetics and enabling Fe3C-Fe1/CNT nanofibers to exhibit excellent performance in H_2_-O_2_ fuel cells and Al-air batteries.

## INTRODUCTION

Exploring new generation energy storage and conversion technology is crucial for the sustainable development of human society [1]. Proton exchange membrane fuel cells (PEMFCs) and metal-air batteries, known for their high energy efficiency and environmentally friendly features, hold great potential for sustainable energy applications [2]. Oxygen reduction reaction (ORR) electrocatalysts are essential for the development of these technologies, such as metal-air batteries and PEMFCs [3–6]. However, the ORR exhibits slow kinetics and often relies on costly and rare platinum group metals (PGMs) as catalysts, which poses a significant bottleneck due to their high cost and limited availability, hindering large-scale application [7–9]. Consequently, there has been considerable interest in developing precious-metal–free catalysts as alternatives to expensive PGM-based ORR electrocatalysts [10–14]. Transition metal/nitrogen co-doped carbon-based materials (M–N–C) have garnered particular attention as emerging non–precious-metal catalysts due to their high intrinsic activity, well-defined active sites, and good stability [15–18]. Specifically, Fe–N–C catalysts, where iron is coordinated with nitrogen in an Fe–N_4_ configuration, demonstrate Pt-like behavior in O_2_ adsorption and the subsequent O=O bond breaking during the ORR catalytic process [19–21]. Nevertheless, the unsatisfactory adsorption energy of Fe–N_4_ sites for oxygen intermediates has led to limited intrinsic activity and severe degradation, especially in acidic media [2,22].

Various strategies to enhance the intrinsic activity of Fe-based catalysts include doping heteroatoms (such as S, P, etc.) into the carbon matrix, introducing neighboring M–N_4_ sites (where M = Co, Ni, Mn, etc.), and constructing axial coordination groups at the metal active center (such as O, OH, Cl, etc.) [23–27]. Particularly, transition metal compounds modified with atomically dispersed Fe–N_4_ catalysts are considered promising candidates to improve ORR performance and stability in acidic media [20]. However, the modulation of the intrinsic activity of Fe–N_4_ sites in acidic media by transition metal compounds remains poorly understood. For instance, the critical impact of water activation on the mechanism, thermodynamics, and kinetics of electrocatalytic reactions has been largely overlooked. Additionally, the strong electric field polarization effect under acidic conditions aligns interfacial water molecules around the metal center into an O-down configuration [28]. This alignment disrupts hydrogen bonding between oxygenated intermediates and interfacial water molecules, impeding proton transfer in the proton-coupled electron transfer (PCET) step and ultimately slowing ORR reaction kinetics. Promoting hydrogen bond formation between oxygen-containing intermediates and interfacial water by constructing efficient water activation catalytic sites is an effective strategy to accelerate the PCET process [29]. The role of platinum species in activating water for various electrocatalytic processes in aqueous systems is well-documented [30,31]. Thus, it is hypothesized that Fe_3_C, with a Pt-like electronic configuration, could serve as an efficient catalytic site for water activation in proton generation from water molecules, complementing the traditional Fe–N_4_ site to enhance ORR activity. Moreover, electrospinning has emerged as a fascinating and scalable technique for fabricating energy-functional composite nanofibers, enabling the incorporation of both single atomic and metallic compounds [32].

In this work, we successfully fabricated the Fe_3_C-modified Fe–N_4_ enriched with a carbon nanotube system (Fe_3_C-Fe_1_/CNT) on carbon nanofibers to achieve efficient and stable ORR using a unique electrospinning technology. The obtained catalyst features inter-crosslinked CNT and a three-dimensional (3D) porous network structure, which enhances active site exposure and facilitates the movement of reactants and products into and out of the active sites, as well as electron transport throughout the structural network. Additionally, the strong electronic perturbation of the Fe–N4 active center by Fe_3_C leads to d-orbital electron rearrangement, optimizing the binding energy of intermediates in the rate-determining step. Notably, the Fe_3_C-Fe_1_/CNT exhibits a half-wave potential of up to 0.83 V in acidic media and 0.91 V in alkaline media. Moreover, delocalized electrons can drive the activation of interfacial water, accelerating the entire PCET process. As a result, the Fe_3_C-Fe_1_/CNT demonstrates a high power density of 724 mW cm^–2^ in H_2_-O_2_ fuel cells and a discharge performance of 1.65 V at 1 mA cm^–2^ in Al-air batteries. This study provides guidance for the rational design of highly active electrospun M–N–C electrocatalysts.

## RESULTS AND DISCUSSION

### Design, synthesis and characterization

**Figure 1.**
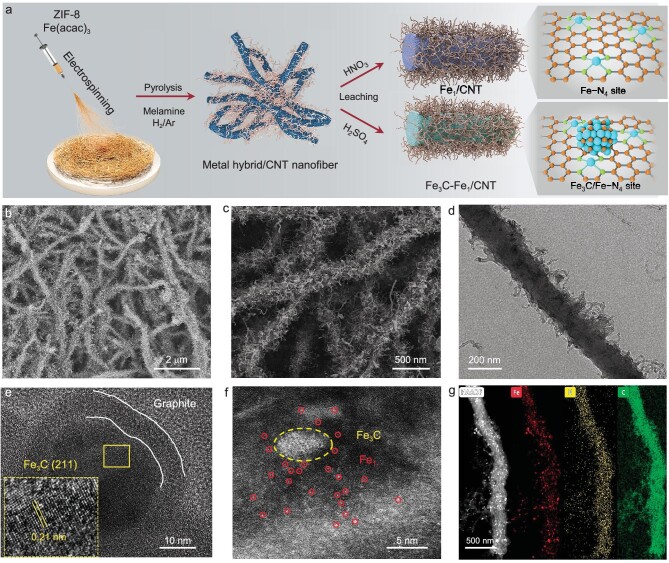
Synthesis and morphological characterization of catalysts. (a) Schematic of the synthesis process of Fe_3_C-Fe_1_/CNT and Fe_1_/CNT. (b–c) SEM images of Fe_3_C-Fe_1_/CNT. (d) TEM and (e) HRTEM images of Fe_3_C-Fe_1_/CNT. (f) AC-HAADF-STEM image of Fe_3_C-Fe_1_/CNT and (g) the corresponding EDS maps of Fe, N, C.

The fabrication approach of Fe_3_C-Fe_1_/CNT is described in Fig. 1a. Typically, the precursor solution was electrospun into a three-dimensional (3D) membrane composed of Fe-embedded 1D carbon nanofibers (Fe-CNF, Fig. S1 in the online Supplementary data). After that, pre-oxidation was performed to stabilize the microstructure and avoid the fusion of the carbon fiber in the subsequent carbonization process. Finally, thermal treatments and acid etching were implemented to form Fe_3_C nanoparticles (NPs) and atomically dispersed Fe-anchored N-doped CNT. Incorporating CNT roughened the carbon nanofiber surface, enhancing active site exposure and facilitating ORR electron/ion transport, while melamine-free heat treatment yielded a smooth surface devoid of CNT structures (Fig. 1b and c, Fig. S2) [33]. The transmission electron microscopy (TEM) images in Fig. 1d and e further confirm the graphite-coated Fe_3_C NPs loading on carbon fiber intimate contact with the bamboo-like CNT structures in Fe_3_C-Fe_1_/CNT. No NPs are observed for Fe_1_/CNT revealing that Fe_3_C NPs are removed after the HNO_3_ acid leaching and the Fe species present in an atomically dispersed state (Fig. S3). Aberration-corrected high-angle annular dark-field STEM (AC-HAADF-STEM) is suitable for further detecting the status of metal species. As shown in Fig. 1f, the carbon substrate contains both Fe_3_C NPs and Fe atomic sites. In addition, the corresponding energy-dispersive X-ray spectroscopy (EDS) shows that the Fe, N, and C elements are distributed uniformly in Fe_3_C-Fe_1_/CNT nanofibers (Fig. 1g). The above results demonstrate that the electrospinning process, thermal treatment, and acid etching route successfully produce the CNT structure, in which Fe_3_C NPs and atomically dispersed Fe sites co-exist in the carbon matrix.

**Figure 2.**
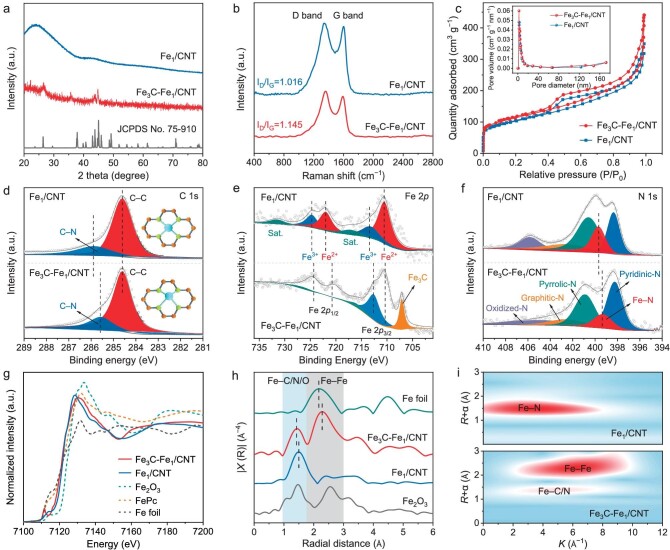
Fine structure characterization of the active sites. (a) XRD patterns. (b) Raman spectra. (c) BET spectra. XPS spectra of (d) C 1s, (e) Fe 2p, (f) N 1s. (g) Normalized Fe K-edge XANES spectra. (h) Fourier transform k^3^-weighted Fe K-edge EXAFS spectra. (i) Wavelet transform of Fe_3_C-Fe_1_/CNT and Fe_1_/CNT.

The catalysts are analyzed using X-ray diffraction (XRD) patterns to determine their chemical makeup and crystal structure. As illustrated in Fig. 2a, the typical Fe_3_C-Fe_1_/CNT diffraction peaks at around 26.6° are attributed to the graphitic carbon structures’ (002) plane. Insignificant Fe_3_C-related diffraction peaks are seen from 42–46° (JCPDS No. 75–910), indicating that a significant portion of the Fe-containing species was removed after H_2_SO_4_ treatment. After further treatment with HNO_3_, the Fe_3_C peaks vanish, leaving just the typical graphitic carbon peak in Fe_1_/CNT [34], which suggests the graphitic carbon is well retained, whereas the NPs are being leached off. For the other control group, distinct graphitized carbon peaks are observed in Fe_3_C-Fe_1_/CNT compared to Fe_3_C-Fe_1_, indicating that the formation of CNT enhances electron transport and corrosion resistance. (Fig. S4). The D-band peak for lattice defects at 1345 cm^−1^ and the G-band peak for sp^2^-hybridized carbon at 1590 cm^−1^ are present for all samples observed by Raman spectroscopy and are analyzed in Fig. 2b. The D-band and G-band peak intensity ratios of Fe_3_C-Fe_1_/CNT (I_D_/I_G_ ≈1.145) are larger than those of Fe_1_/CNT (I_D_/I_G_ ≈1.016). This demonstrates that the carbon matrix of Fe_3_C-Fe_1_/CNT contains many more carbon nanostructures with defects, which can cooperate with Fe–N–C active centers and enhances electronic conductivity, modulates local electron density, facilitates oxygen intermediate adsorption, and improves electron transfer kinetics, thereby significantly boosting overall catalytic efficiency and performance. The Brunauer–Emmett–Teller (BET) specific surface area of Fe_3_C-Fe_1_/CNT is measured by isothermal N_2_ adsorption-desorption analysis (Fig. 2c). The specific surface area of Fe_3_C-Fe_1_/CNT is 399.7 m^2^ g^–1^, higher than that of Fe_1_/CNT (363.4 m^2^ g^–1^). Fe_3_C-Fe_1_/CNT shows many mesopores during pyrolysis due to the presence of Zn atoms, facilitating the penetration and transport of reaction products during electrocatalysis. X-ray photoelectron spectroscopy (XPS) is per performed to investigate the electron and coordination structure of catalysts (Fig. 2d–f, Fig. S5). The C–N in Fe_3_C-Fe_1_/CNT experiences a decrease in binding energy, suggesting that the electronic structure of the Fe–N_4_ symmetry is disrupted and transferable to the carbon substrate. The peak at 706.8 eV is ascribed to Fe with mixed valence states suggesting the existence of Fe_3_C, which may activate the carbon layer outer surface to promote its involvement in electrocatalysis [35]. In comparison with the Fe_1_/CNT, the Fe–N species in Fe_3_C-Fe_1_/CNT show a shift toward lower binding energy. These results evidence that strong electronic perturbation of Fe_3_C NPs lead to an asymmetric distribution of Fe–N_4_ electron density. Significantly, Fe_3_C-Fe_1_/CNT exhibits larger contents of pyridinic-N, Fe–N, and graphitic-N species (Table S1). Pyridinic-N may lower the energy barrier for O_2_ to adsorb on nearby carbon atoms, which can behave as an efficient active site to speed up the generation of oxygen-containing intermediates in the ORR process [36,37].

Local electronic changes can be further observed in the fine structure of X-ray absorption (XAFS). The Fe_3_C-Fe_1_/CNT absorption edge falls between the absorption edges of FePc and Fe_2_O_3_, indicating that the oxidation state of Fe lies in the range of +2 to +3, that is, Fe^2+^ and Fe^3+^ coexist in the Fe_3_C-Fe_1_/CNT catalyst (Fig. 2g). In Fe–N–C catalysts, only the Fe^3+^–N_4_ structure is generally obtained [38]. One of the main reasons here is that during the material synthesis process, Fe^2+^ in the iron source is easily oxidized to Fe^3+^ to form the Fe^3+^–N_4_ structure [39]. In fact, the Fe^2+^–N_4_ structure is much more catalytically active than the Fe^3+^–N_4_ structure for ORR [40]. Furthermore, Fe_3_C-Fe_1_/CNT displays a negative absorption edge compared to Fe_1_/CNT, suggesting that the Fe center has a reduced valence and a larger electron density. The presence of more electronegative groups like Fe_3_C has an impact on the active center of Fe_1_/CNT, according to the positive absorption edge compared to FePc. Thus, the introduction of Fe_3_C leads to the conversion of Fe^3+^ to Fe^2+^ and the asymmetric distribution of Fe–N_4_ electron density, which is consistent with the XPS analysis. The Fe K-edge Fourier-transformed extended XAFS (FT-EXAFS) spectrum provides significant structural information (Fig. S6, Table S2). As shown in Fig. 2h, the peak position contributed to Fe–N of Fe_3_C-Fe_1_/CNT in *R*-space is significantly negatively shifted, which implies a shortening of the Fe–N bond length and a change in the local structure of the Fe–N_4_ active sites [41,42]. The Fe–Fe bonds are represented by peaks in Fe_3_C-Fe_1_/CNT at 2.2 Å, which is consistent with the presence of Fe_3_C species [43]. Wavelet-transform EXAFS (WT-EXAFS) of the corresponding catalysts are performed (Fig. 2i, Fig. S7). The WT-EXAFS analyses of Fe_1_/CNT show the greatest intensity at ∼1.51 Å and 3.57 Å^−1^, confirming the coordination structure of Fe–N. In contrast, Fe_3_C-Fe_1_/CNT show maximum peak intensity at shorter 1.41 Å and longer 4.88 Å^–1^, suggesting the superposition of the Fe–N bond and Fe–C bond. Combining XPS and XANES/EXAFS analyses, we successfully obtained a composite structure of Fe_3_C/Fe–N_4_ with an asymmetric distribution of the electron density of Fe–N_4_ around the Fe active center.

### ORR performance evaluation of Fe_3_C-Fe_1_/CNT

**Figure 3.**
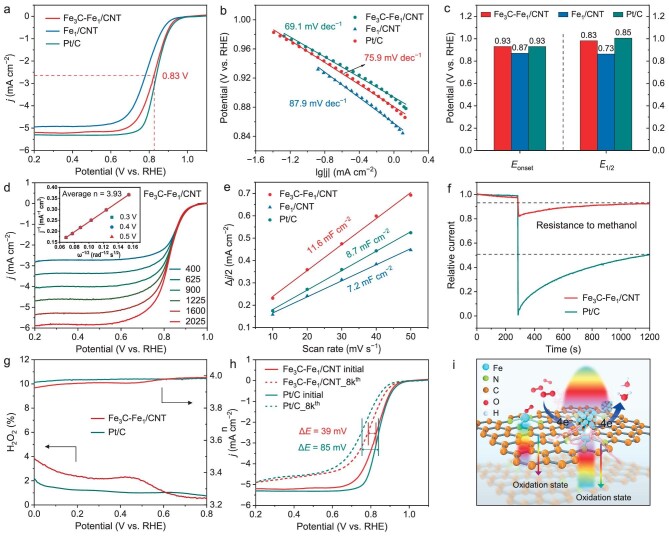
Electrocatalytic activity evaluation. (a) LSV curves of Fe_3_C-Fe_1_/CNT, Fe_1_/CNT, and 20% Pt/C in 0.5 M H_2_SO_4_. (b) Tafel slopes for the corresponding catalysts. (c) *E*_onset_ and *E*_1/2_. (d) ORR polarization curves of Fe_3_C-Fe_1_/CNT at different rotating sweeps. Inset: the fitted K-L plots and electron transfer numbers. (e) Plots of current densities (at 0.04 V vs. RHE) as functions of scan rates. (f) Methanol tolerance of the catalysts in 0.5 M H_2_SO_4_. (g) H_2_O_2_ yield and electron transfer number of the catalysts. (h) Stability tests. (i) Schematic representation of ORR properties versus oxidation state.

The electrocatalytic activity of each resulting catalyst toward the ORR was assessed utilizing a conventional three-electrode rotating ring-disk electrode (RRDE) system in the O_2_-saturated 0.5 M H_2_SO_4_. The cyclic voltammetry (CV) curves unmistakably display a reduction peak, while the N_2_-saturated solution lacks a matching peak (Fig. S8), proving the electrochemical response of Fe_3_C-Fe_1_/CNT toward the ORR. The linear sweep voltammetry (LSV) tests are performed to evaluate the catalysts ORR activity (Fig. 3a). The ultimate Fe_3_C-Fe_1_/CNT exhibits a Tafel slope of 75.9 mV dec^−1^, which is similar to that of Pt/C and smaller than Fe_1_/CNT (Fig. 3b). Fe_3_C-Fe_1_/CNT exhibits an onset potential (*E*_onset_) of 0.93 V and a half-wave potential (*E*_1/2_) of 0.83 V, which is preferable to Fe_1_/CNT, and somewhat lesser activity than Pt/C catalyst (Fig. 3c). In addition, the resulting Fe_3_C-Fe_1_/CNT ORR activity is substantially better than most of the previously reported M–N–C catalysts (Table S3). The Koutecky-Levich (*K–L*) equations might be used to determine the number of electrons transported (*n*) (Fig. 3d, Fig. S9). The *n* value for Fe_3_C-Fe_1_/CNT is determined to be 3.93 according to the linearity of the *K–L* plots in the potential of 0.3 V, 0.4 V, 0.5 V. Compared to Fe_1_/CNT, Fe_3_C-Fe_1_/CNT exhibits a higher double-layer capacitance, which is correlated with its electrochemical surface area (Fig. 3e, Fig. S10), leading to improved electrochemical performance. Additionally, no current degradation is seen after adding methanol to the electrolyte, which displays remarkable methanol resistance (Fig. 3f). As shown in Fig. 3g, the H_2_O_2_ yields stay below 5%, indicating a 4-electron ORR route over the Fe_3_C-Fe_1_/CNT. After 8000 CV cycles, the *E*_1/2_ of Fe_3_C-Fe_1_/CNT only negatively shifts 39 mV, whereas the 85 mV negative shifts for Pt/C (Fig. 3h). As a result, Fe_3_C NPs adorned Fe–N_4_ with higher electron cloud density are more successful than the Fe–N_4_ sites in lowering the ORR reaction energy barrier, which results in a lower overpotential (Fig. 3i).

Fe_3_C-Fe_1_/CNT not only performs well in acid solution but also exhibits remarkable ORR performance in 0.1 M KOH solution. Fe_3_C-Fe_1_/CNT with an *E*_1/2_ of 0.91 V outperforms other catalysts in terms of alkaline ORR activity (Figs S11 and S12), and the majority of M–N–C electrocatalysts described thus far (Table S4). In addition, as shown in Figs S13–S15, the Fe_3_C-Fe_1_/CNT exhibits high selectivity for 4-electron ORR, the excellent stability and resistance to methanol. Fe_3_C-Fe_1_/CNT was also used as the Al-air battery cathode catalyst given the optimized electrocatalytic activity for alkaline ORR. The solid electrolyte with a polyacrylic acid (PAA) base facilitates flexibility while concurrently offering sufficient mechanical support (Fig. S16a). The Fe_3_C-Fe_1_/CNT based Al-air battery can sustain a discharge voltage of 1.65 V for 28 hours at a discharge current density of 1 mA cm^–2^, which is higher than that of Pt/C, Fe_1_/CNT and the majority of solid-state Al-air batteries that have been previously documented (Fig. S16b, Table S5). Fe_3_C-Fe_1_/CNT produces a greater voltage than Fe_1_/CNT, particularly during elevated discharge current densities, as demonstrated by the rate performance measurement (Fig. S16c). Due to the electron transfer process occurring more quickly, discharge polarization shows that Fe_3_C-Fe_1_/CNT may accomplish a peak power density of 44 mW cm^–2^, which is higher than Fe_1_/CNT (26 mW cm^–2^), Pt/C (28 mW cm^–2^), and outperform most of their cathode catalyst counterparts’ all-solid-state Al-air batteries (Fig. S16d–f).

### PEMFC performance evaluation of Fe_3_C-Fe_1_/CNT

**Figure 4.**
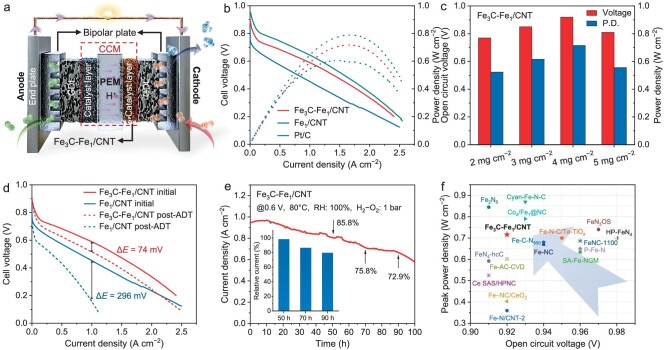
H_2_-O_2_ PEMFC performance. (a) Structure illustration of the H_2_-O_2_ fuel cell. (b) Polarization and power density curves by using the catalysts as cathodes. (c) Comparison of different loads of Fe_3_C-Fe_1_/CNT. (d–e) Stability tests. (f) Comparison of PEMFC performance with reported advanced catalysts.

To further explore the application potential of Fe_3_C-Fe_1_/CNT, we assemble a PEMFC to assess efficacy and stability (Fig. 4a, Fig. S17). With Fe_3_C-Fe_1_/CNT catalyst loading of 4 mg cm^−2^ shows an open-circuit voltage of 0.91 V, close to the 0.94 V of the Pt/C-based cell at the usual 1.0 bar H_2_/O_2_ at 80°C. Additionally, the PEMFC could produce current densities of 1.39 A cm^−2^ at 0.5 V and 2.40 A cm^−2^ at 0.2 V (Fig. 4b). The Fe_3_C-Fe_1_/CNT-assembled PEMFC exhibits high performance with a peak power density of 716 mW cm^−2^, significantly higher than that of Fe_1_/CNT (517 mW cm^−2^). Despite showing comparable ORR-catalytic activity on RRDE, Fe_3_C-Fe_1_/CNT and Pt/C catalysts exhibit contrasting behaviors in PEMFC. Fe_3_C particle sintering may disrupt Nafion-ionomer distribution and diminish the effectiveness of the cathode's three-phase reaction interface. Variations in catalyst structure, including Fe loading and pore size distribution, contribute to noticeable current density differences between Fe_3_C-Fe_1_/CNT and Fe_1_/CNT. It is evident that when metal loading rises, peak power density increases initially and then declines, reaching its maximum value at 4 mg cm^−2^ (Fig. 4c). A suitable loading could ensure that electrocatalytic activity develops sufficiently since high loading results in an increasingly thick catalyst layer that may obstruct mass transportation and charge transmission. Polarization plots before and after an aggressive square-wave accelerated durability test (ADT) further documented cell voltage loss (Fig. 4d). Once more, the Fe_1_/CNT assembled PEMFC exhibits the greatest performance deterioration, with a cell voltage loss of 296 mV, which is significantly higher than that of Fe_3_C-Fe_1_/CNT (74 mV loss). Significantly, Fe_3_C-Fe_1_/CNT based PEMFC could discharge steadily for 100 hours at 0.6 V, demonstrating the well-durability (Fig. 4e). As shown in Fig. 4f and Table S6, the Fe_3_C-Fe_1_/CNT exhibited excellent stability and competitive activity levels compared with previously reported Fe–N–C catalysts. No significant changes in the valence states of the corresponding elements were observed in Fe_3_C-Fe_1_/CNT after ADT, as determined by XPS analysis (Fig. S18). Furthermore, the morphology was well maintained and no particle aggregation was detected, and the uniform distribution of Fe_3_C nanoparticles and Fe single atoms was well-preserved, as evidenced by the AC-HAADF-STEM image obtained post-ADT (Fig. S19), confirming the retention of the highly stable ordered structure. The foregoing superior performance and durability qualities point to the Fe_3_C-Fe_1_/CNT potential for application in fuel cells.

### The origin of enhanced activity of Fe_3_C-Fe_1_/CNT

**Figure 5.**
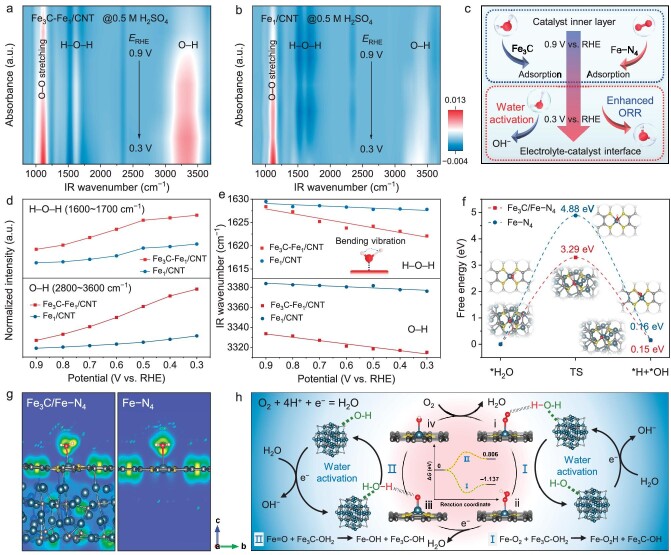
*In-situ* characterization and reaction mechanism. (a–b) *In situ* FTIR spectra of the Fe_3_C-Fe_1_/CNT and Fe_1_/CNT catalysts for ORR. (c) Scheme of the structure-activity-potential relationship. (d) The detailed variations of H−O−H and O−H with potential. (e) The variations of wavenumber with potential. (f) The energy configuration of water activation on the catalysts. (g) ELF evaluations for H_2_O adsorption on the Fe site of the catalysts. (h) ORR mechanism of water activation for Fe_3_C-Fe_1_/CNT.

To verify our conjecture, *in situ* Fourier transform infrared (FTIR) spectroscopy measurements were used to probe the interfacial water structure and further explore the water activation mechanism. As shown in Fig. 5a and b, O−H stretching modes (∼2800–3600 cm^−1^) and H−O−H bending modes (∼1600–1700 cm^−1^) of interfacial water can be observed, which provides clear evidence for the involvement of surface H_2_O in the ORR. The H−O−H and O−H stretching strength at each potential in Fe_3_C-Fe_1_/CNT is much larger than Fe_1_/CNT, indicating that the Fe_3_C sites have a significant affinity for H_2_O activation and form interfacial hydrogen bonds (Fig. 5d) [44]. More importantly, the O−H stretching peaks at each potential in Fe_3_C-Fe_1_/CNT are mainly located near 3330 cm^−1^ redshifted by 50 cm^−1^ compared with Fe_1_/CNT (∼3380 cm^−1^), suggesting a strong hydrogen-bonding interaction between the interfacial water molecules and the ORR oxygen intermediates [29]. The H_2_O bending vibration has a higher wave number on Fe_3_C-Fe_1_/CNT than on Fe_1_/CNT, showing that the presence of Fe_3_C strengthens interfacial hydrogen bonding (Fig. 5e) [45]. The *in-situ* experimental results confirmed the mechanism of water activation (Fig. 5c), which was further investigated in-depth through density functional theory (DFT) calculations. The transition state (TS) formation on Fe_3_C/Fe–N_4_ and Fe–N_4_ in the hydrolysis and dissociation process is endothermic, confirming it as the rate-determining step (RDS). In this RDS, the Δ*G* on Fe_3_C/Fe–N_4_ is 3.29 eV, significantly smaller than that on Fe–N_4_ at 4.88 eV (Fig. 5f). The remarkably low energy barriers in RDS explain the outstanding activity of Fe_3_C/Fe–N_4_ in water activation and the facile dissociation of water into protons for the ORR. Further insights into the water interface mechanism were gained through theoretical calculations, including evaluations of the electron localization function (ELF) to measure excess kinetic energy density due to Pauli repulsion [46]. As a result, compared to Fe–N_4_, Fe_3_C/Fe–N_4_ shows enhanced polarization in the O−H bonding of adsorbed H_2_O, illustrating the regulatory influence of Fe_3_C on water molecule bonding states, and suggesting that strong polarity is advantageous for hydrogen dissociation (Fig. 5g). Specifically, O_2_ preferentially binds to the Fe−N_4_ sites to form Fe−N_4_-O_2_. The spontaneous dissociation of O_2_ molecules is aided by interfacial water hydrogen bonding rather than just the presence of Fe−N_4_ sites. H_2_O preferentially binds to the Fe_3_C site to form Fe_3_C-OH_2_ and generates OH^−^ by 1e^–^ reduction. Surface protons can be transferred from Fe_3_C-OH_2_ to neighboring Fe−N_4_-O_2_, leading to the formation of Fe_3_C-OH and Fe−N_4_-OOH, which subsequently undergoes 1e^–^ reduction to produce Fe−N_4_=O and H_2_O. Then Fe−N_4_=O undergoes the same PCET process and returns to the initial state (Fig. 5h). Meanwhile, we observed similar intermediate products in alkaline media, which highlights the pH-universal water activation induced ORR mechanism for our catalyst (Fig. S20). The extraordinary feature of this mechanism includes the interfacial activated water by the turnover of Fe_3_C-OH/Fe_3_C-OH_2_ and the surface proton transfer between the proximate Fe_3_C and Fe−N_4_ sites.

### DFT calculations

**Figure 6.**
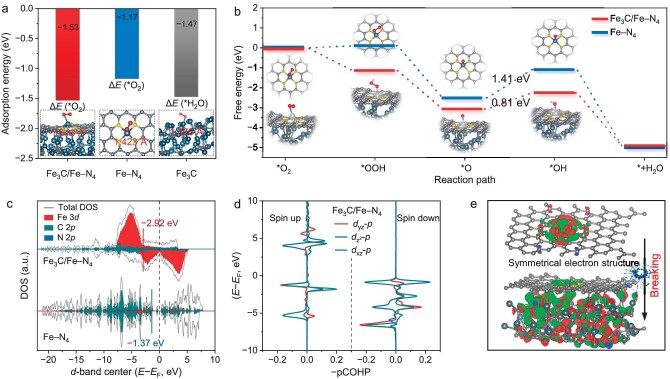
Theoretical calculation of the catalytic activity. (a) Adsorption energies for H_2_O and O_2_. (b) Free energy diagram of ORR steps on Fe_3_C/Fe–N_4_ and Fe–N_4_. (c) DOS of Fe_3_C/Fe–N_4_ and Fe–N_4_. (d) COHP analysis of Fe−O bond after *OH adsorption for Fe_3_C/Fe–N_4_. (e) Schematic representation of the regulatory effect of Fe_3_C toward ΔG_*OH_ on Fe−N_4_ sites. The red and green zones represent the charge accumulation and dispersion, respectively.

In the Fe_3_C/Fe–N_4_ model, O_2_ can be readily adsorbed as well as activated, and the O–O bond has the largest stretching length (Fig. 6a), which implies that the O–O bond is relatively the easiest to break resulting in higher ORR activity [47]. Supported by the above analysis, the optimized models of ORR on Fe_3_C/Fe–N_4_, Fe–N_4_, and Fe_3_C are established with the free energy ladder images (Fig. 6b, Fig. S21). For the Fe–N_4_ and Fe_3_C models, since the production of *OOH and *OH intermediate is endothermic in contrast to the other stages, the development and breakdown of them constitute the RDS of ORR. For the Fe_3_C/Fe–N_4_ model, the free energy values for the *OOH step are spontaneous, implying that from O_2_ to *OOH is more advantageous in terms of thermodynamics. Although the significant adsorption of *OH intermediates on Fe_3_C/Fe–N_4_ model, the energy barrier of *OH on Fe_3_C/Fe–N_4_ (0.81 eV) is lower than Fe–N_4_ (1.41 eV) and Fe_3_C (1.18 eV), which is essential for speeding up the ORR activity. The analysis of the partial density of states (DOS) for O_2_ molecules adsorbed in various models may be employed to assess the degree of binding between reactants and metal centers. The Fe_3_C/Fe–N_4_ model exhibits a Fe *d*-band center energy of −2.92 eV, compared to the Fe–N_4_ (−1.37 eV), indicating weaker interaction and easier dissociation of intermediates (Fig. 6c, Fig. S22). This strong electronic interaction between Fe_3_C species and Fe–N_4_ sites leads to down-shifted *d*-band centers in catalysts, moderating bonding strength of oxygen-related intermediates and enhancing ORR activities.

To elucidate active site mechanisms, the projected density of states (PDOS) of Fe 3*d* orbitals (*d*_xz_, *d*_xy_, *d*_yz_, ${{d}_{{{{\mathrm{x}}}^{\mathrm{2}}}{\mathrm{ - }}{{{\mathrm{y}}}^{\mathrm{2}}}}}$, and ${{d}_{{{{\mathrm{z}}}^{\mathrm{2}}}}}$) is shown in Fig. S23. Most of the electron orbitals involved in non-homogeneous catalysis concentrate near the Fermi level. In Fe_3_C/Fe–N_4_, ${{d}_{{{{\mathrm{x}}}^{\mathrm{2}}}{\mathrm{ - }}{{{\mathrm{y}}}^{\mathrm{2}}}}}$ is minimally influential, while non-localized *d*_xz_, *d*_yz_, and ${{d}_{{{{\mathrm{z}}}^{\mathrm{2}}}}}$ orbitals show homogeneous Fermi level distribution, underscoring their role in catalytic efficiency. To identify the reason for the high intrinsic catalytic activity center, the *d*_xz_-*p, d*_yz_-*p*, and ${{d}_{{{{\mathrm{z}}}^{\mathrm{2}}}}}$-*p* were further computed using projected crystal orbital Hamilton population (pCOHP). For Fe–N_4_, significant localized electronic effects are observed in the *d*_yz_-*p* and ${{d}_{{{{\mathrm{z}}}^{\mathrm{2}}}}}$-*p* bonding orbitals and spin-down antibonding orbitals, respectively, and exhibit unusually high intensity (Fig. S24). As a result, the intermediations are more affected by the very localized enhancing interaction, which makes it challenging to dissociate. On the contrary, Fe_3_C/Fe–N_4_ could observe significant electron delocalization. As shown in Fig. 6d, the *d*_xz_-*p, d*_yz_-*p*, and ${{d}_{{{{\mathrm{z}}}^{\mathrm{2}}}}}$-*p* have non-localized enhancement interactions in spin up or spin down, meaning that the *O is easily formed into *OH by the moderately intense localized enhancement interaction acting on it. Consequently, a greater limiting potential (0.93 V) is observed for Fe_3_C-Fe_1_/CNT in experimental results. The Fe_3_C NPs altered the electronic arrangement of the Fe center orbitals on the Fe−N_4_ site, thereby disrupting the symmetric electronic structure of the Fe−N_4_ site and significantly altering the degree of electronic delocalization of the spin up and spin down in the *d*_xz_-*p, d*_yz_-*p*, and ${{d}_{{{{\mathrm{z}}}^{\mathrm{2}}}}}$-*p* orbitals enhancing the Fe−O bonding strength (Fig. 6e). Thus, based on *in situ* FTIR as well as DFT, results indicate that the increased activity at ORR-related potentials is mainly dependent on the dissociation of interfacial water induced by delocalized electrons, thereby strengthening the hydrogen bonding interactions of interfacial water molecules with ORR oxygen intermediates.

## CONCLUSION

In summary, we successfully constructed Fe_3_C-modified, atomically dispersed Fe-N co-doped CNT anchored on nanofibers using an electrospinning-pyrolysis-etching route. The strong electronic perturbation of the Fe−N_4_ active center by Fe_3_C leads to electronic rearrangement in the Fe center orbitals, enhancing electron delocalization in the *d* orbitals and achieving an asymmetric electron density distribution at the Fe−N_4_ sites. Delocalized electrons can activate the dissociation of water molecules and accelerate PCET processes by forming hydrogen bonds with surface oxygen intermediates. The Fe_3_C-Fe_1_/CNT catalyst demonstrated a high power density of 716 mW cm^–2^ in H_2_-O_2_ fuel cells and a high discharge performance of 1.65 V at 1 mA cm^–2^ in Al-air batteries. This strategy may inspire the development of more efficient single-atom electrocatalysts, providing a new and insightful perspective on improving the inherent catalytic performance of electrospun M−N−C catalysts.

## Supplementary Material

nwae193_Supplemental_File
