# Supplementary material for: Fe-N co-doped carbon nanofibers with Fe3C decoration for water activation induced oxygen reduction reaction
Source: Natl Sci Rev. 2024 Jun 4;11(10):nwae193. doi: 10.1093/nsr/nwae193 (PMC11409866; doi:10.1093/nsr/nwae193)
Supplement: nwae193_Supplemental_File [file nwae193_supplemental_file.pdf]

Supporting Information

Fe-N co-doped carbon nanofibers with Fe_3C decoration for water activation induced oxygen reduction reaction

Shaoxiong Li[1], *Gengyu Xing*[1], *Sheng Zhao*[1], *Jian Peng*[2], *Lingfei Zhao*[2], *Feng Hu*[1], *Linlin Li*[1], *Jiazhao Wang*[2], *Seeram Ramakrishna*[3], *and Shengjie Peng*[1,*]

[1]College of Materials Science and Technology, Nanjing University of Aeronautics and Astronautics, Nanjing 210016, China;

[2]Institute for Superconducting and Electronic Materials Australian Institute for Innovative Materials, University of Wollongong Innovation Campus, North Wollongong, NSW 2522, Australia;

[3]Department of Mechanical Engineering, National University of Singapore, Singapore 117583, Singapore

*Corresponding author. E-mail: pengshengjie@nuaa.edu.cn

1. Experimental Section

1.1 Synthesis

Synthesis of ZIF-8. ZIF-8 crystals were prepared via rapidly pouring an aqueous solution (16 mL deionized water) of $Zn(NO_3)_2$ $6H_2O$ (7.9 mmol, 2.34 g) into an aqueous solution (160 mL deionized water) of 2-methylimidazole (553 mmol, 45.4 g). Then, the mixed solution was magnetically stirred for 5 minutes at room temperature. After stirring, the product was collected by centrifugation (7000 rpm for 30 min) and thoroughly cleaned with deionized water at least five times. Finally, the product was dried at 60 ℃ for 12 h in vacuum.

Synthesis of Fe_3C-Fe_1/CNT. A mixture of 0.8 g $Fe(acac)_3$, 0.8 g ZIF-8, and 0.8 g of polyacrylonitrile (PAN) was dissolved in 10 mL of N, N-dimethylformamide (DMF) solution with stirring to obtain a homogeneous solution. After stirring overnight, a viscous precursor solution was loaded into a plastic syringe with a stainless-steel nozzle, which was connected to a high-voltage power supply. A high voltage of 18 kV was applied between the needle tip and aluminum collector, and the spinning rate was controlled at 1 mL h^{-1}. Then, the spun fibers were sintered at 250 ℃ for 2 h in the air. Afterward, melamine (molar ratio 1:10) was added and pyrolyzed for 1 h at 900 ℃ in 5 % H_2/Ar atmosphere. Finally, 100 mg of the sample was added to 50 mL of 0.5 M H_2SO_4 and stirred at 80 ℃ for 10 h to obtain Fe_3C-Fe_1/CNT. The Fe_1/CNT was prepared with the same procedure as Fe_3C-Fe_1/CNT, only using 5 M HNO_3 acid to leach.

Synthesis of Fe_3C-Fe_1. The synthesis process of Fe_3C-Fe_1 is identical to that of Fe_3C-Fe_1/CNT, with the exception that melamine is not included during the annealing step in a hydrogen-argon mixture.

1.2 Characterizations

X-ray diffraction (XRD) was measured on the Bruker D8 ADVANCE with Cu Kα (λ=1.54178 Å) radiation. Scanning electron microscope (SEM) images were taken on JEOL JSM-7600F. Transmission electron microscope (TEM) was taken on FEI Tecnai G2 F20 equipped with an energy-dispersive X-ray spectroscopy analyzer. Aberration-corrected scanning transmission electron microscope (AC STEM) images were taken at JEM-ARM200F equipped with JED-2300T SDD operated at 200 kV. Powder X-ray photoelectron spectroscopy (XPS) analysis

used Al Kα as an exciting radiation source on an Escalab 250Xi system. The extended X-ray absorption fine spectroscopy (EXAFS) was tested at Taiwan Photon Source (TPS) beamline, 44A Quick-scanning X-ray absorption spectroscopy (XAS), in National Synchrotron Radiation Research Center (NSRRC), Hsinchu, Taiwan.

1.3 Electrochemical measurements

All electrochemical measurements were conducted in a conventional three-electrode configuration with an Autolab PGSTAT302N electrochemical workstation. The rotating disk or ring disk glassy carbon electrode, KCl saturated Ag/AgCl electrode, and graphite rod are used as working electrodes, reference electrodes, and counter electrodes, respectively. To prepare the working electrode, 5 mg of the catalyst was dispersed into 950 μL ethanol and 50 μL Nafion under ultrasonication for 30 min to form the uniform ink. After that, 10 μL of the prepared ink was dropped on the working electrode and dried naturally. 0.1 M KOH electrolyte was bubbled with O_2 or N_2 for 30 min before the measurement. The electron transfer number (*n*) and H_2O_2 yield (%) were respectively calculated by the following equations.

$$n = \frac{4\ \mathrm{ID}}{ID+(IR/N)} \qquad (1)$$

$$\mathrm{H_2O_2}\ \% = \frac{2\ \mathrm{IR/N}}{\mathrm{ID+(IR/N)}} \qquad (2)$$

I_D is the Faradaic current at the disk, I_R is the Faradaic current at the ring, and N is the H_2O_2 collection coefficient at the ring.

1.4 Solid-state flexible Al-air batteries assemble

The solid-state flexible Al-air battery employed a sandwich-type structure. Pre-cleaned carbon cloth was used to support the air-cathode, and a 0.1 mm thick Al foil (>99.99 wt%) was used as the anode. The polyacrylic acid-based polymer was utilized as a gel electrolyte. To make the gel electrolyte, three separate solutions were prepared first. (1) The 10 mL aqueous solution containing 6.497 g KOH, 6.104 mg of ZnO, 21.269 mg of Na_2SnO_3, and 0.829 mg of $In(OH)_3$ was prepared as an alkaline solution. (2) A polymer solution was prepared by dissolving 0.15 g N, N'-methylene-bis-acrylamide (MBA) in a 1 g acrylic acid (AA) solution. (3) The polymerization initiator was prepared by dissolving 2 g $K_2S_2O_8$ in 10.5 ml deionized water, with a concentration of 16 wt.%. Secondly, the prepared alkaline solution was mixed with the polymer solution and kept stirring for 5 min. The white precipitation formed during this process was filtered out using

filter paper. Then, 100 μL of the pre-prepared polymerization initiator was added to the remaining clear solution to form the gel electrolyte. 200 μL of the ORR ink was cast onto a cleaned carbon cloth with an active surface area of 1 cm^2 to work as an air cathode. All solid-state battery measurements were performed under ambient conditions.

1.5 Assembly and test of PEMFC

Typically, the Fe_3C-Fe_1/CNT catalyst powder as cathode was dispersed in Nafion- ionomer solution and then sprayed onto one side of a Nafion 211 membrane to fabricate the catalyst-coated membrane (CCM). The other side was covered by the 60 % PtRu/C catalyst with a loading of 0.9 mg_{PtRu} cm^{-2} as anode. The electrode area was 5 cm×5 cm. Different loadings of Fe_3C-Fe_1/CNT catalyst in the cathode were studied to find the optimal value. Finally, the prepared cathode and anode were pressed onto the two sides of a Nafion 211 membrane at 135 ℃ for 300 seconds under a pressure of 2 MPa to obtain the membrane-electrode assembly (MEA). The H_2-O_2 fuel cells were tested under standard 1.0 bar H_2/O_2 gases at 100 % relative humidity and 80 ℃ in galvanic mode; the flow rates of both H_2 and O_2 gases were 300 mL min^{-1}. The square-wave accelerated durability test (ADT) included 30,000 cycles of square wave with each cycle holding the MEA at a voltage of 0.6 V for 3 s and then 0.95 V for 3 s.

1.6 Computational Details

The theoretical calculation was conducted in the density functional theory (DFT) framework implemented in the Vienna ab initio package (VASP). The Perdew-Burke-Ernzerhof (PBE) with generalized gradient approximation (GGA) was utilized as an exchange-correlation functional. The ionic core was described by the projected augmented wave (PAW) method. The energy cutoff was set at 500 eV. Gaussian smearing method with a width of 0.05 eV was used to allow the partial occupancies of Kohn-sham orbitals. Sampling the Brillouin zone used a k-point grid of 3×3×1. The electronic structure iteration convergence criterion was set to 10^{-5} eV. All structures were optimized until the energy difference was less than 0.02 eV $Å^{-1}$. Grimme's DFT-D2 methodology described dispersion interactions in the adsorption model. We further perform density functional theory (DFT) calculations to explore the active site of Fe_3C/Fe-N_4 for ORR. According to EXAFS analyses, we built Fe_3C-Fe_1/CNT by loading layer (211) surface during the whole calculation process, which is much closer to reality from the analyses of XAS and

HAADF-STEM images. To get a better view of the oxygen and intermediates adsorption state on active sites, we only cut off a small part of the three models. In this structure, Fe and N atoms replace part of the carbon atoms after structural optimization and proceed with a series of calculations. The free energy values are calculated by placing H_2 and H_2O in a 10*10*10 cubic lattice, respectively.

The ORR reaction for four-electron pathway in alkaline media:

(i) $O_2 (g) + * \rightarrow *O_2$

(ii) $* + O_2 (g) + H_2O(l) + e^- \rightarrow *OOH + OH^-$ ΔG1

(iii) $*OOH + e^- \rightarrow *O + OH^-$ ΔG2

(iv) $*O + 2H^+ + 2e^- + H_2O \rightarrow *OH + OH^-$ ΔG3

(v) $*OH + e^- \rightarrow * + OH^-$ ΔG4

Overall reaction:

$O_2 (g) + H_2O(l) + 4e^- \rightarrow 4OH^-$

For each ORR reaction step, the Gibbs free energy of the formation can be calculated by the following equation:

$\Delta G = \Delta E + \Delta ZPE - T\Delta S - eU$

ΔE is the reaction energy of a given reaction step, which can be obtained from the DFT calculation. ΔZPE and ΔS are the corrected zero-point energy and entropy, respectively. U is the applied potential vs RHE, and e is the transferred charge. The Gibbs free energy of oxygen is calculated according to the following formula, Where the experimental reaction energy is 4 × 1.23 eV = 4.92 eV.

$O_2 (g) + 2H_2(g) \rightarrow H_2O(l)$

Thus, $G_{O2} = 2G_{H2O} - 2G_{H2} + 4 \times 1.23$(eV).

$G(_{OH}^-) - G(_e^-)$ can be calculated by the following formula

$2H_2O(l) + 4e^- \rightarrow 2OH^- + H_2(g)$

Thus, $G(_{OH}^-) - G(_e^-) = 2G_{H2O} - 1/2G_{H2} + pH{*}k_BT\ln \alpha_H{}^+$

The mechanism of ORR involves the formation processes of the *OH, *O, and *OOH intermediates. For ORR processes, the ideal thermodynamic free energy change of the intermediates should be closed to zero, indicating that no energy would be wasted.

The η for ORR is calculated based on the following formula:

η_{ORR} = max {ΔG1, ΔG2, ΔG3, ΔG4}/e + 1.23V

2. Supplementary Figures

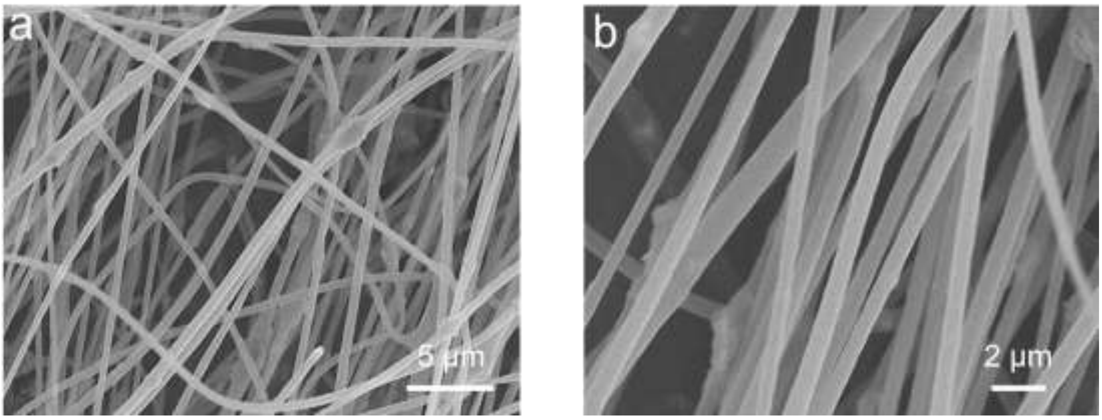

Figure S1. (a) Low magnified and (b) high magnified SEM images of the precursor after electrospinning.

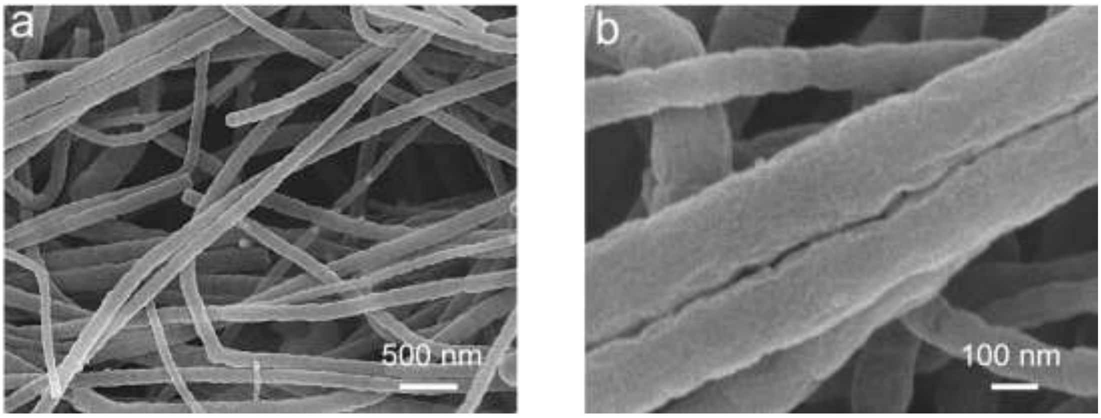

Figure S2. (a) Low magnified and (b) high magnified SEM images of comparative sample without melamine addition.

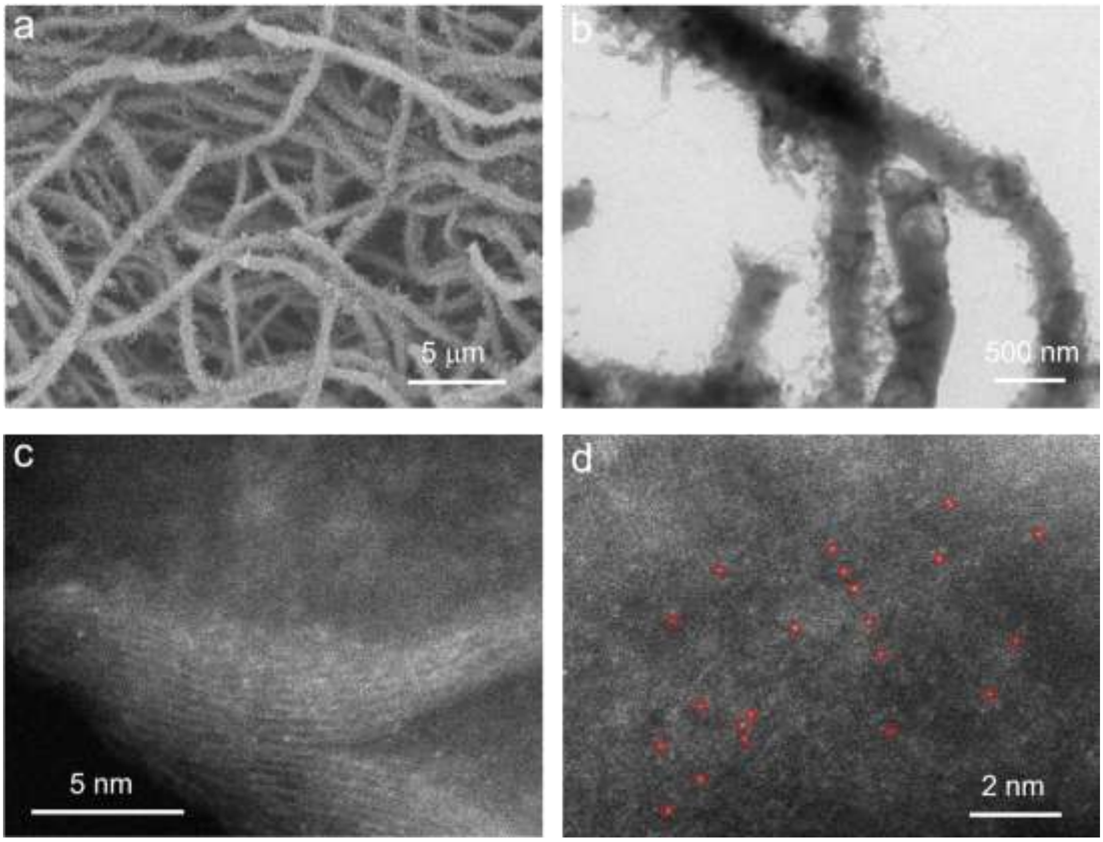

Figure S3. (a) SEM image of Fe_1/CNT. (b) TEM image of Fe_1/CNT. (c-d) HAADF-STEM image of Fe_1/CNT.

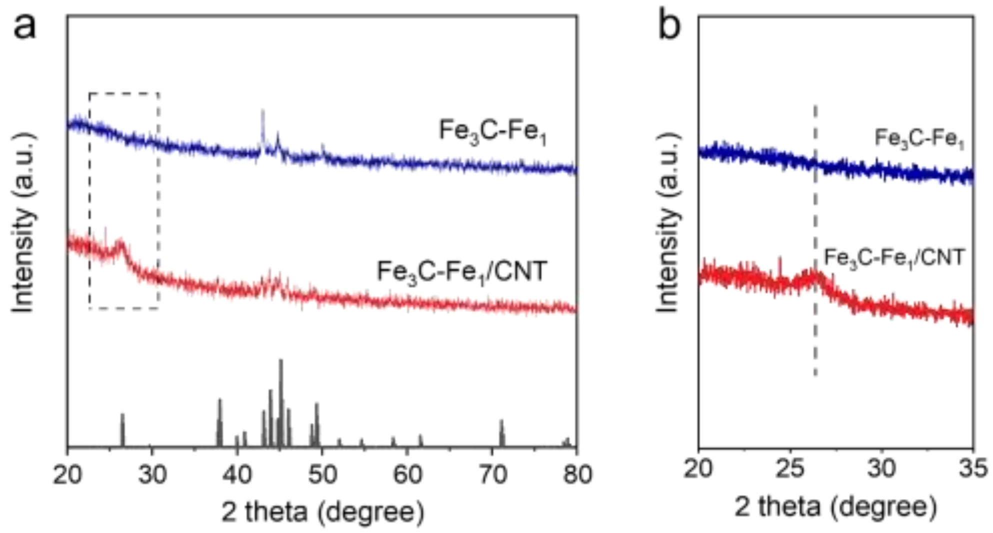

Figure S4. XRD patterns of Fe_3C-Fe_1/CNT and Fe_3C-Fe_1.

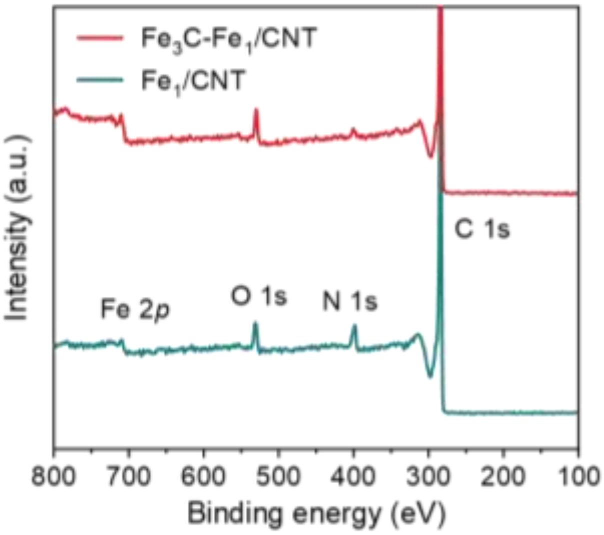

Figure S5. XPS survey spectra of Fe_3C-Fe_1/CNT and Fe_1/CNT.

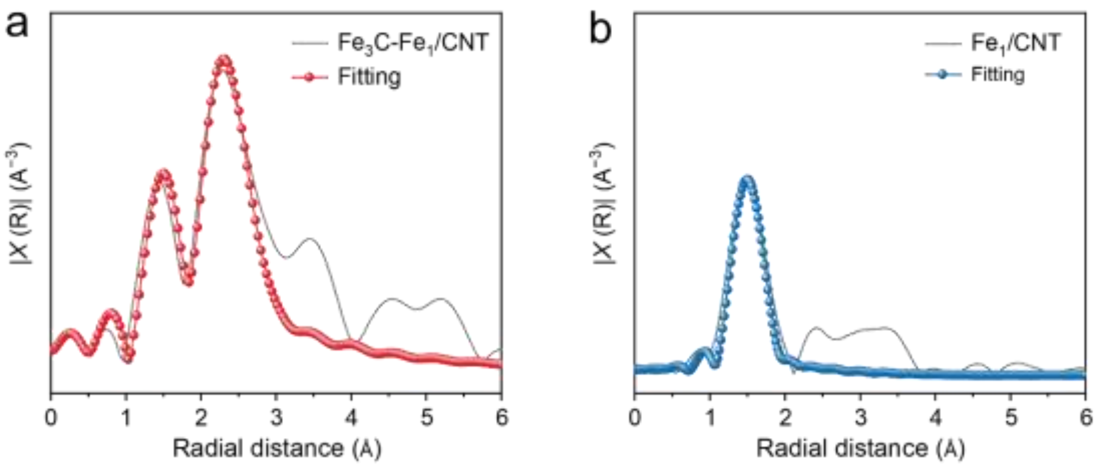

Figure S6. Fitting of the EXAFS spectrum of (a) Fe_3C-Fe_1/CNT and (b) Fe_1/CNT.

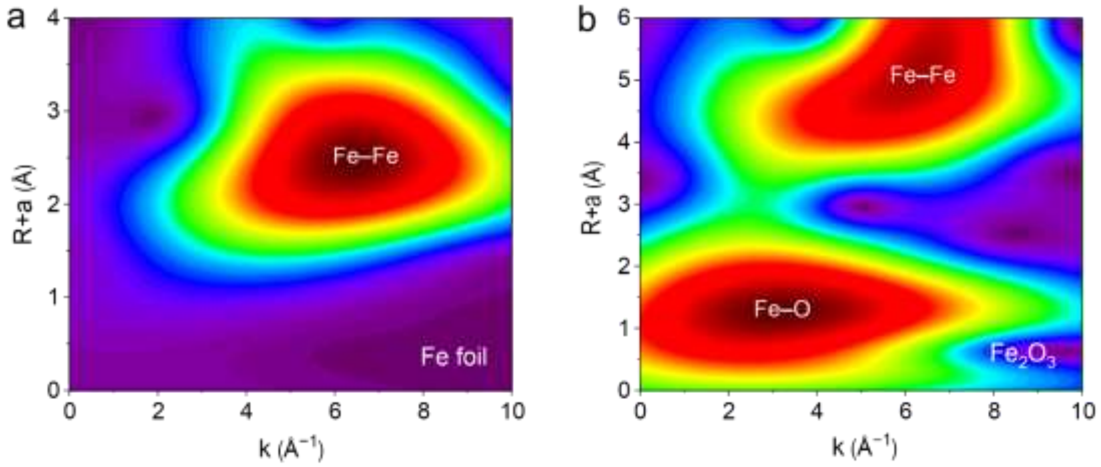

Figure S7. Wavelet transform of (a) Fe foil and (b) Fe_2O_3.

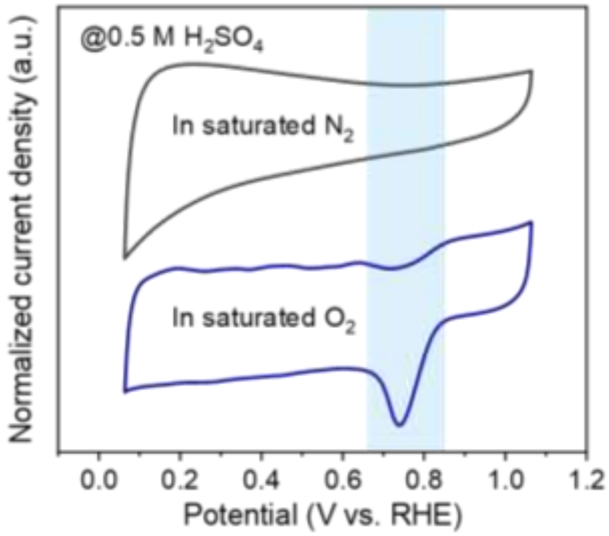

Figure S8. CV curves of Fe_3C-Fe_1/CNT in N_2- and O_2-saturated 0.5 M H_2SO_4 at a scan rate of 50 mV s^{-1}.

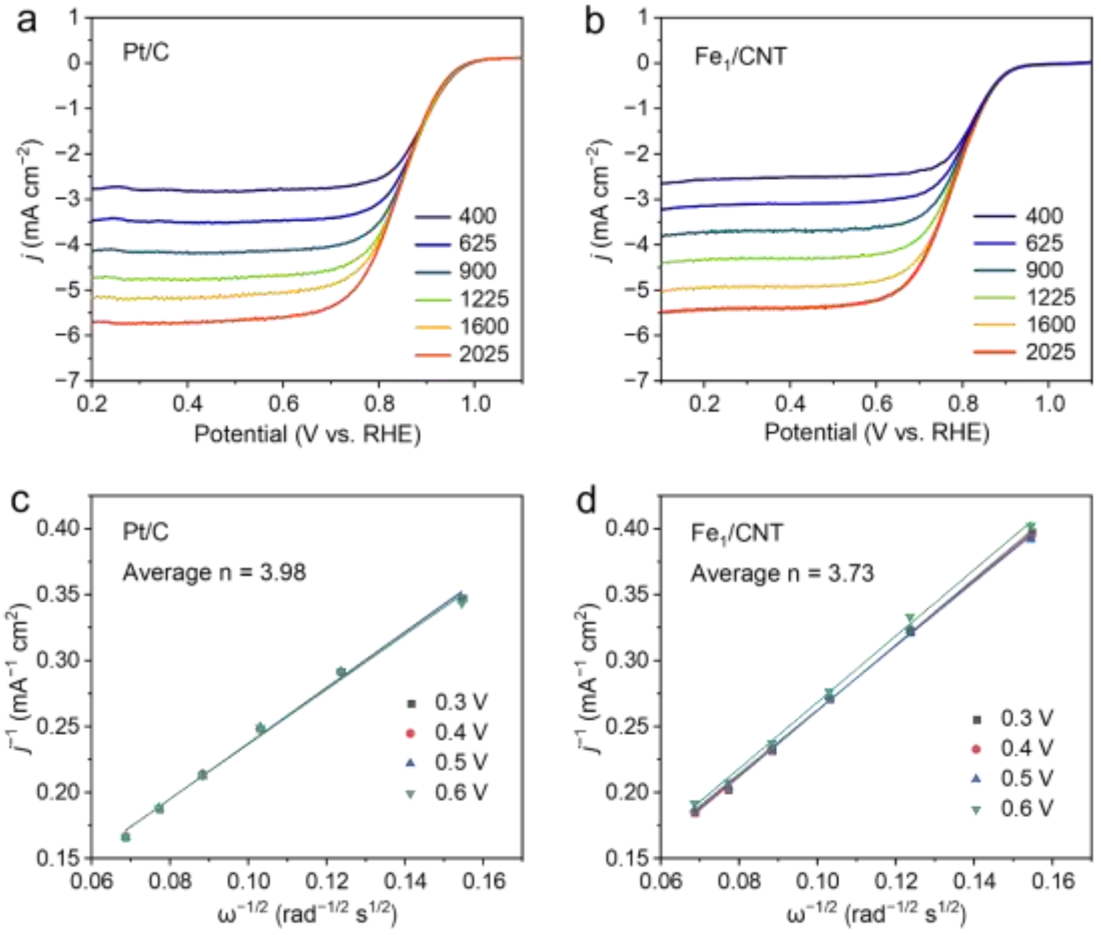

Figure S9. (a-b) ORR polarization curves of Pt/C and Fe_1/CNT at different rotating sweeps in 0.5 M H_2SO_4. (c-d) The fitted K-L plots of the corresponding catalysts.

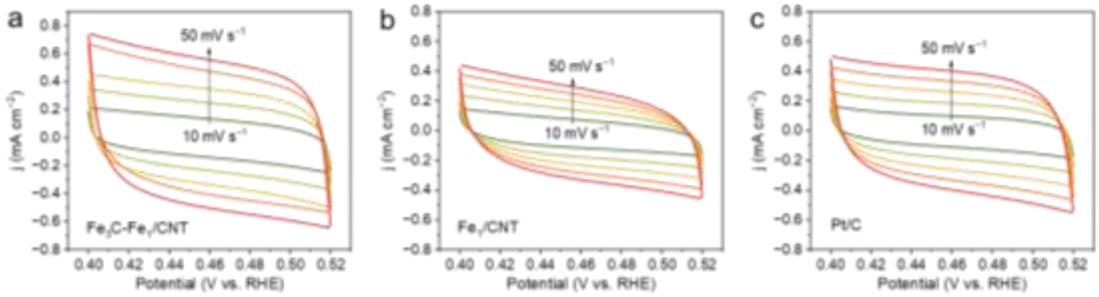

Figure S10. CV measurements of (a) Fe_3C-Fe_1/CNT, (b) Fe_1/CNT, and (c) Pt/C under different scan rates, respectively.

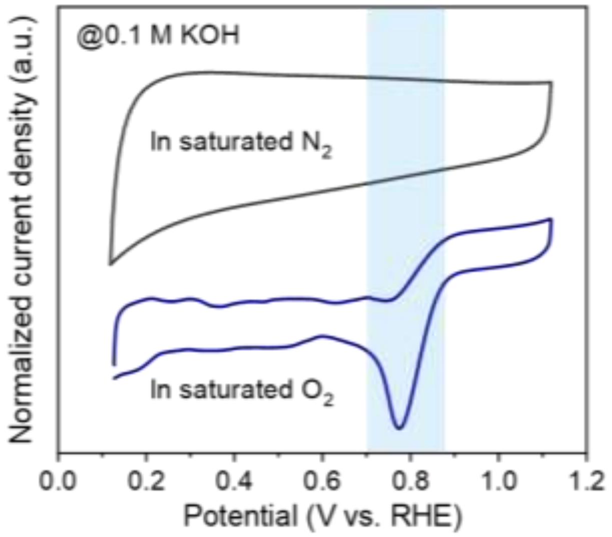

Figure S11. CV curves of Fe_3C-Fe_1/CNT in N_2- and O_2- saturated 0.1 M KOH at a scan rate of 50 mV s^{-1}.

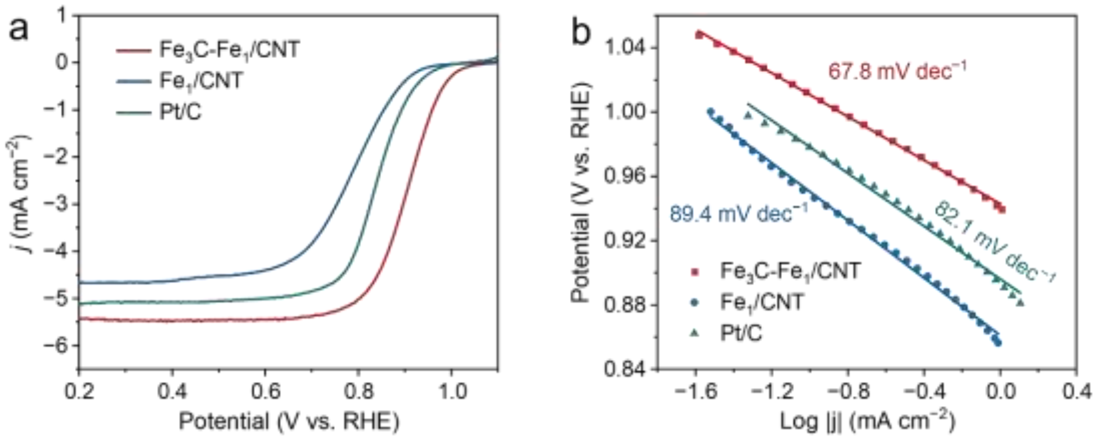

Figure S12. (a) RRDE LSV curves of Fe_3C-Fe_1/CNT, Fe_1/CNT, and 20% Pt/C with a scanning rate of 5 mV s^{-1} at 1600 rpm in 0.1 M KOH. (b) Tafel slope for the resultant catalysts.

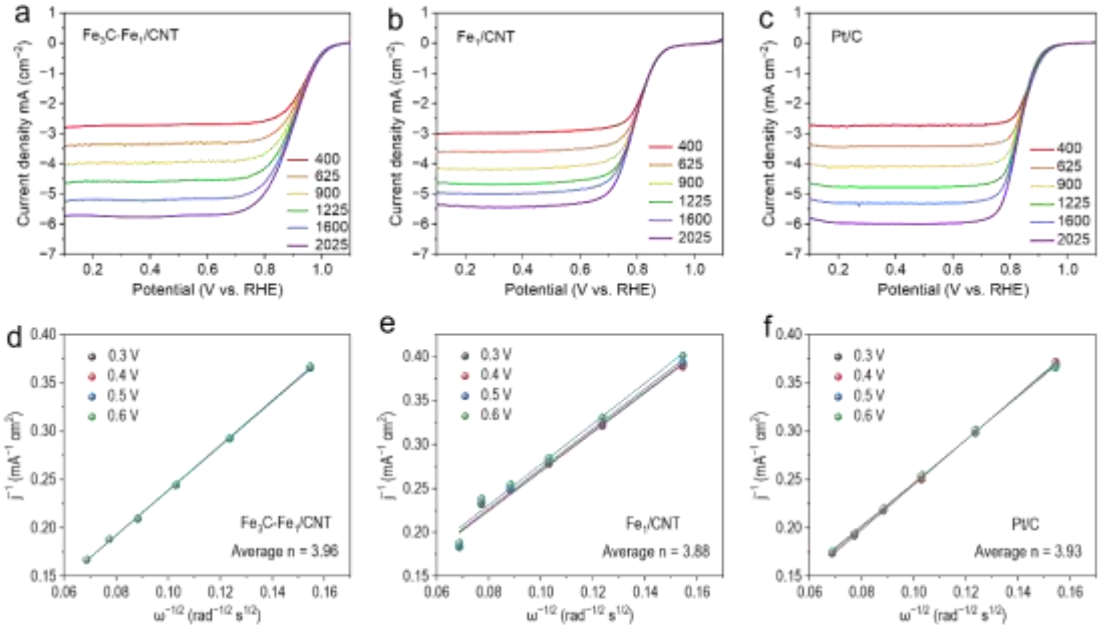

Figure S13. (a-c) LSV curves of Fe_3C-Fe_1/CNT, Fe_1/CNT, and Pt/C at different rotating speeds in 0.1 M KOH. (d-f) The fitted K-L plots of the resultant catalysts.

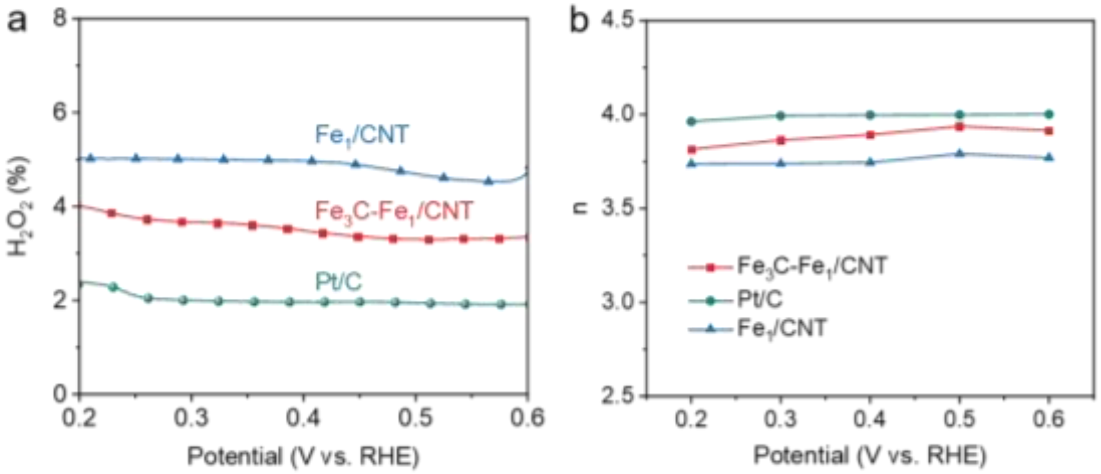

Figure S14. (a) H_2O_2 yield and (b) electron transfer number of Fe_3C-Fe_1/CNT, Fe_1/CNT, and Pt/C.

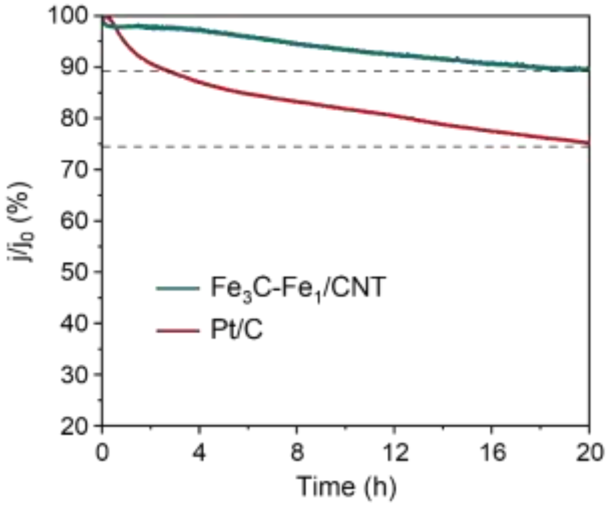

Figure S15. Stability tests of Fe_3C-Fe_1/CNT and Pt/C in 0.1 M KOH.

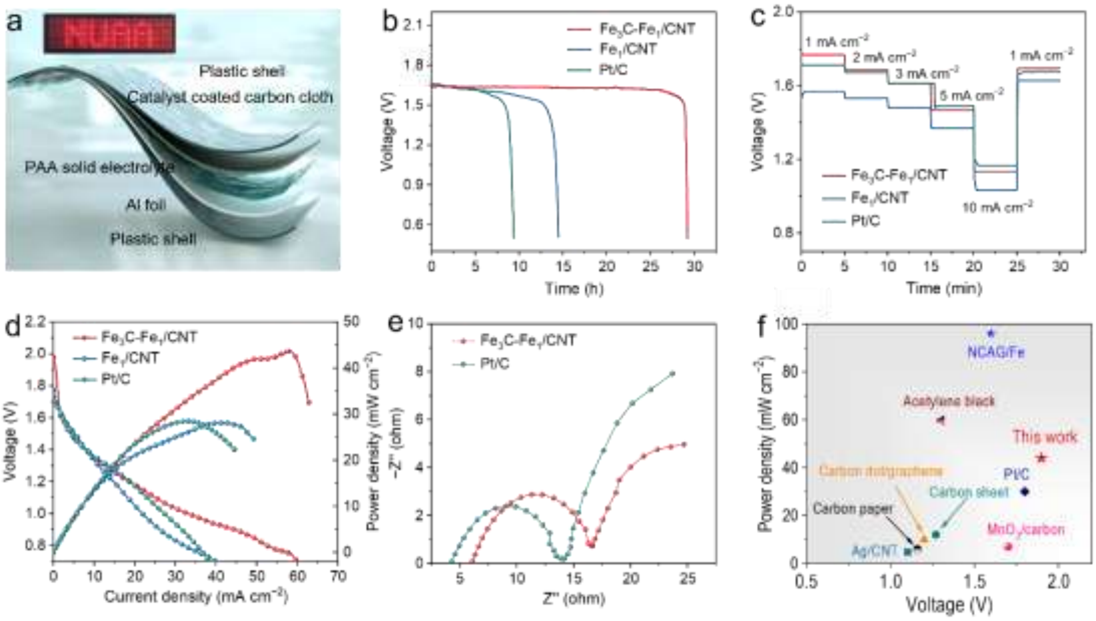

Figure S16. (a) Solid-state Al-air battery structure schematic. (b) Galvanostatic discharge curves. (c) Rate discharge curve. (d) Discharge polarization curves and corresponding power density curves of the samples. (e) EIS comparison. (f) Comparison of activity performance between this work and recent studies.

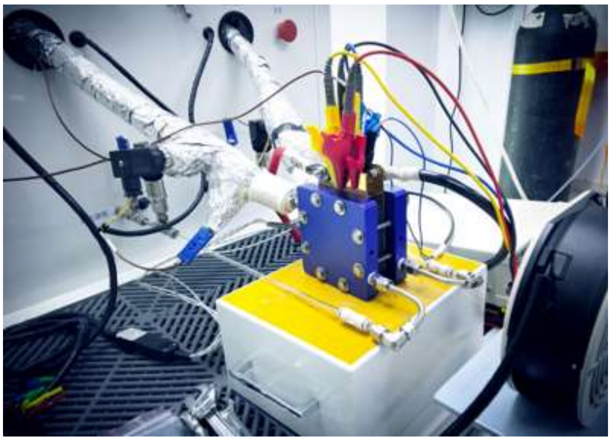

Figure S17. Optical photograph showing a component of a PEMFC with an active area of 25 cm^2

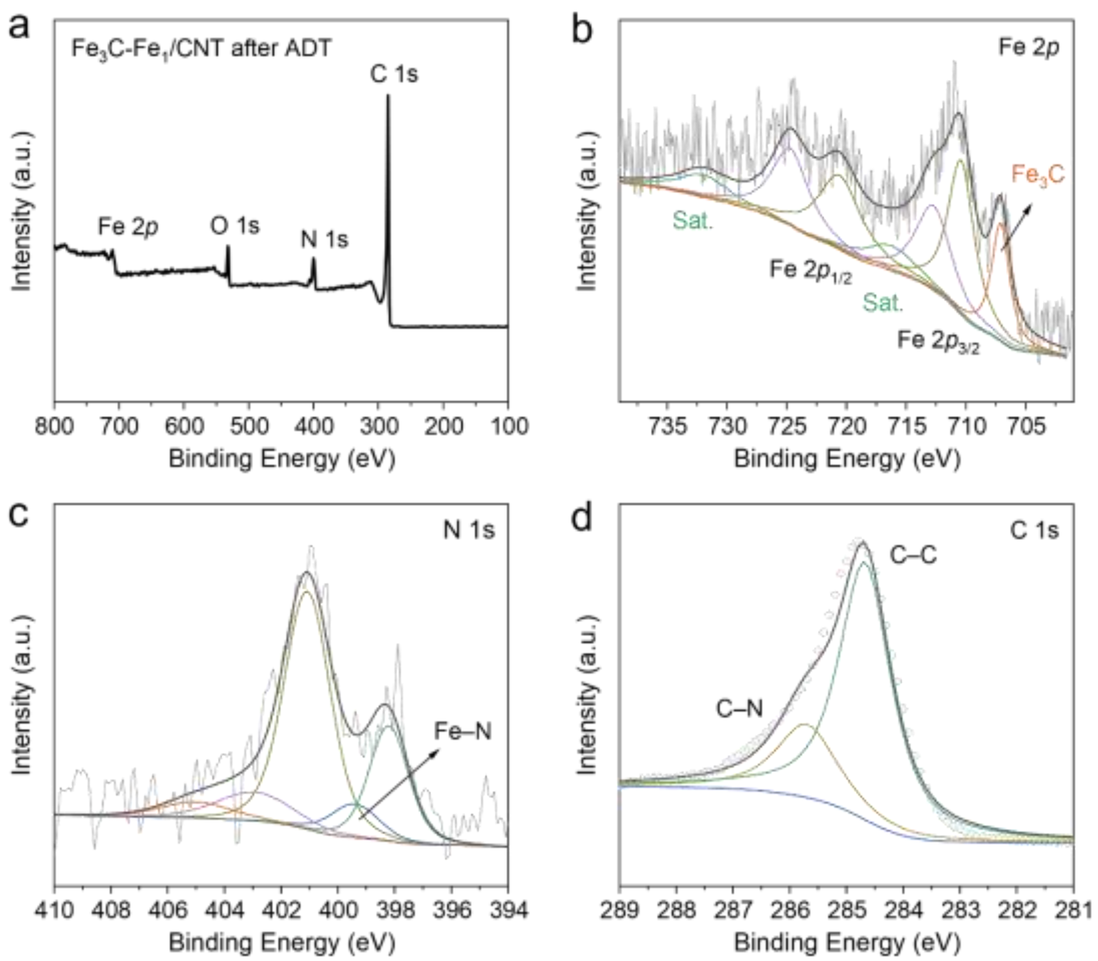

Figure S18. (a) XPS survey spectra of post-ADT Fe_3C-Fe_1/CNT. XPS fine spectra of (b) Fe 2p, (c) N 1s, and (d) C 1s, respectively.

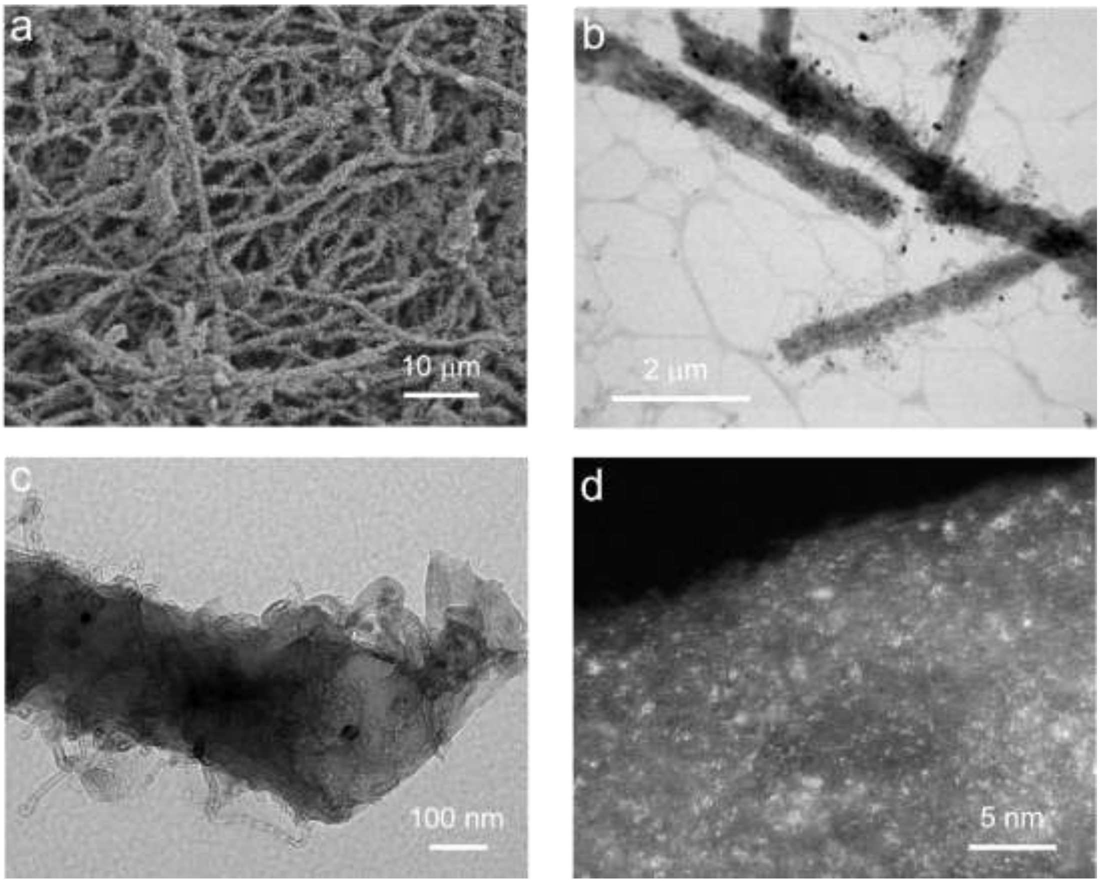

Figure S19. (a) SEM image (b) TEM image (c) HRTEM image and (d) AC-HAADF-STEM of post-ADT Fe_3C-Fe_1/CNT

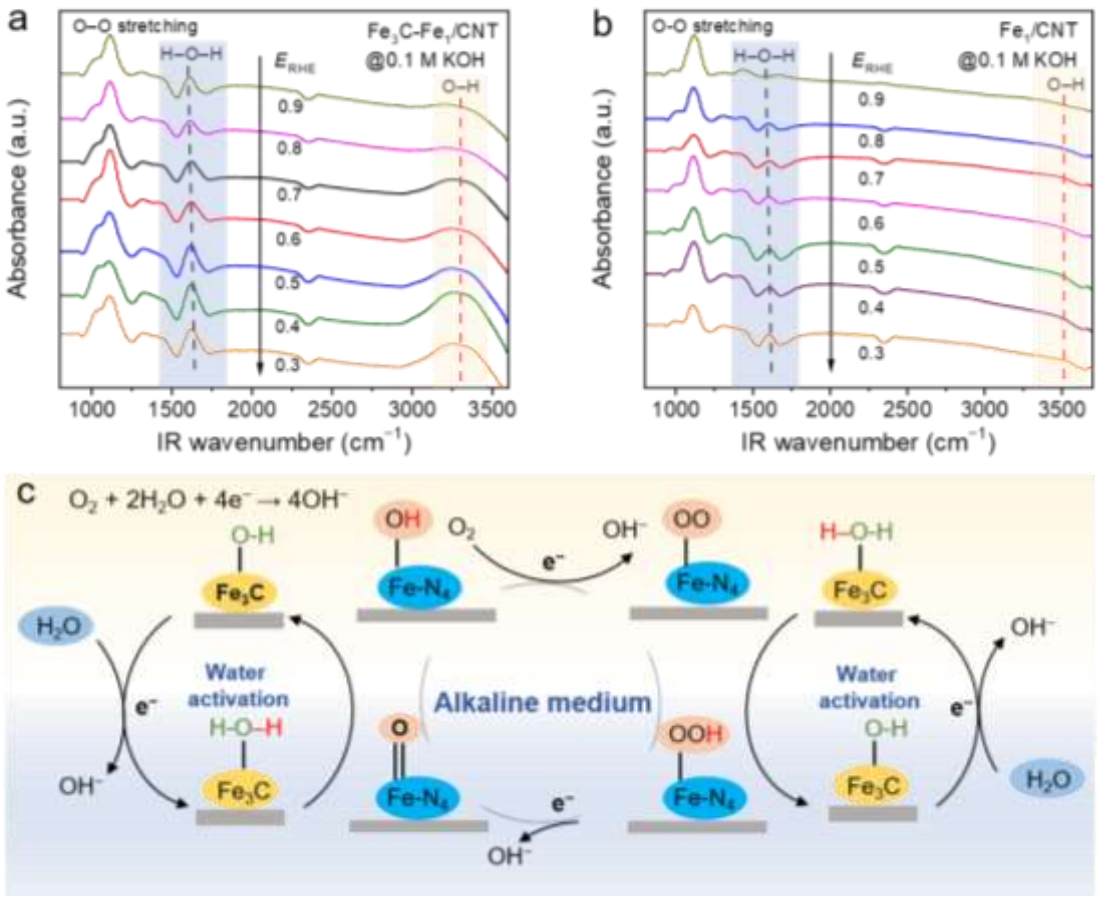

Figure S20. (a-b) In situ FTIR spectra for Fe_3C-Fe_1/CNT and Fe_1/CNT in 0.1 M KOH. (c) Alkaline ORR mechanism of water activation for Fe_3C-Fe_1/CNT.

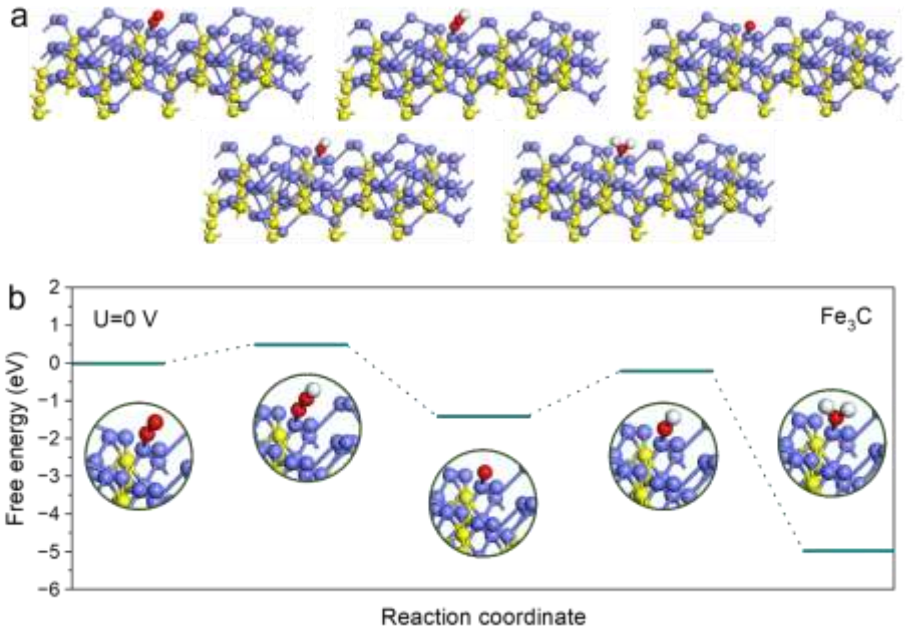

Figure S21. (a) The optimized structures of the model adsorbed *OOH, *O, and *OH, respectively (from the left). Yellow, white, red, and blue represent C, H, O, and Fe atoms, respectively. (b) Free energy diagram for ORR process on the Fe_3C model at the U = 0 V vs RHE, pH = 0.

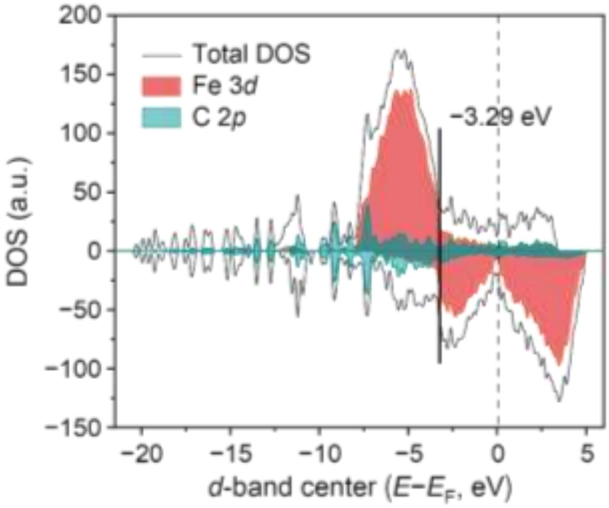

Figure S22. DOS of Fe_3C model.

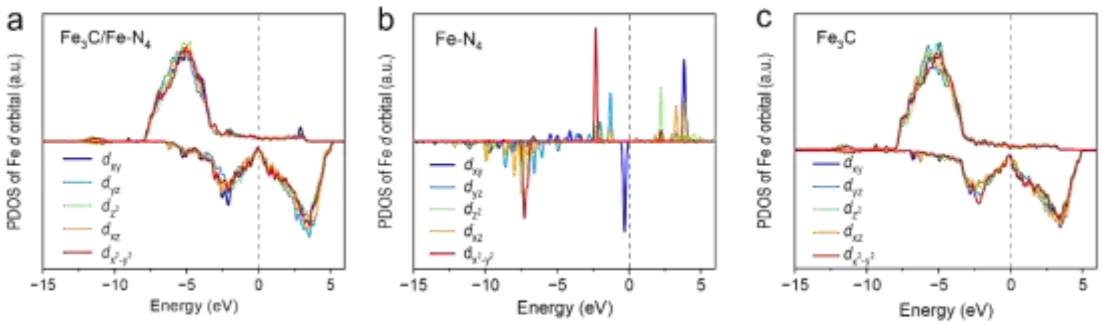

Figure S23. (a-c) Projected Fe 3*d* orbital density of states for Fe_3C/Fe−N_4, Fe−N_4 and Fe_3C.

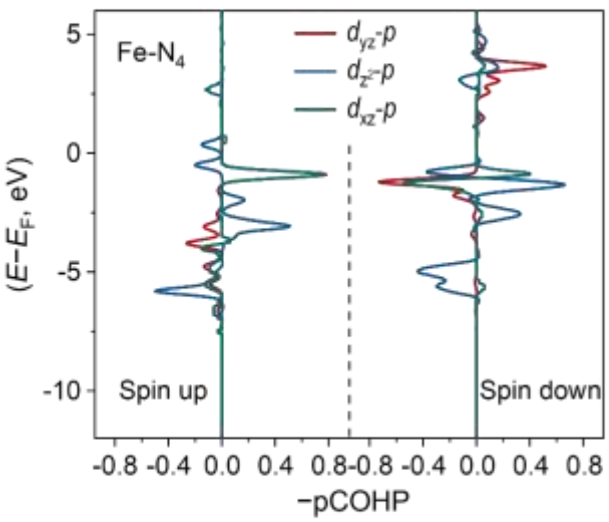

Figure S24. COHP analysis of Fe−O bond after *OH adsorption for Fe–N_4.

3. Supplementary Tables

Table S1. The content of different N types for the prepared catalysts is calculated from XPS.

Samples	Pyridinic-N	Pyrrolic-N	Oxidized-N	Graphitic-N	Fe−N
Fe_3C-Fe_1/CNT	28.4	23.1	11.6	16.1	20.8
Fe_1/CNT	26.6	23.7	14.8	15.5	19.4

Table S2. FT-EXAFS fitting data results of Fe_3C-Fe_1/CNT and Fe_1/CNT.

Samples	Bond	CN	R (Å)	ΔE_0 (eV)	R factor
Fe_3C-Fe_1/CNT	Fe−N	4.1 ±0.3	1.87 ±0.01	-6.9 ±0.03	0.0012
	Fe−Fe	8.0 ±0.2	2.51 ±0.03	-5.4 ±0.03	
Fe_1/CNT	Fe−N	4.4 ±0.1	1.92 ±0.02	-4.6 ±0.32	0.0061

Table S3. Comparison of ORR performance of Fe_3C-Fe_1/CNT in this work with other reported transition-metal-based single-atom catalysts in acidic media.

Catalysts	$E_{1/2}$	E_{onset}	Reference
**Fe_3C-Fe_1/CNT**	**0.83**	**0.93**	**This work**
COPBTC@Cl-CNTs	0.75	0.89	Angew. Chem. Int. Ed. 2023, 62, e202305441
Fe-SA/PNC	0.83	0.95	Angew. Chem. Int. Ed. 2023, 62, e202307504
Fe5-Cu-N-mC	0.80	0.95	Angew. Chem. Int. Ed. 2023, 62, e202308344
FeMnac/MnN_4C	0.78	0.89	Angew. Chem. Int. Ed. 2023, 59, 14988
Fe-NC	0.79	0.85	Angew. Chem. Int. Ed. 2020, 59, 13923
Fe-N-C	0.83	0.93	Adv. Mater. 2023, 35, 2302666
Cyan-Fe-N-C	0.83	0.92	Adv. Mater. 2023, 35, 2305945
Co-COP/AFGC	0.72	0.88	Adv. Mater. 2022, 2, 2208661
Co/Fe-N@CHC	0.81	0.87	Adv. Mater. 2021, 33, 2104718
H@FeNC	0.80	0.88	Adv. Mater. 2021, 33, 2103600
SA-Fe-NGM	0.83	0.93	J. Am. Chem. Soc. 2022, 144, 9280
NDC-Fe-HT	0.80	0.89	J. Am. Chem. Soc. 2021, 143, 18010
Co-N/C-MnO	0.66	0.85	Adv. Funct. Mater. 2022, 2210143
4.2-FeSA	0.74	0.89	Energy Environ. Sci. 2022, 15.1183
FeCo-N-HCN	0.75	0.86	Adv. Funct. Mater. 2021, 31, 2011289
COP-Ppcfe	0.74	0.87	Nat. Commun. 2022, 13, 57
FeSA-N-C	0.80	0.88	Nat. Commun. 2020, 11, 2831
Fe-SA-NSFC	0.82	0.92	Nat. Commun. 2020, 11, 5892
FeSA/FeAC-2DNPC	0.81	0.94	Nat. Commun. 2022, 13, 2963

Table S4. Comparison of ORR performance of Fe_3C-Fe_1/CNT in this work with other reported transition-metal-based single-atom catalysts in alkaline media.

Catalysts	$E_{1/2}$	E_{onset}	Reference
**Fe_3C-Fe_1/CNT**	**0.91**	**0.98**	**This work**
FePc-NT	0.88	0.96	Angew. Chem. Int. Ed. 2023, 62, e202309545
CoTAA-Cl@GR	0.82	0.91	Angew. Chem. Int. Ed. 2023, 135, 16667
Fe-SA/PNC	0.92	1.04	Angew. Chem. Int. Ed. 2023, 62, 17504
Pz-FeTPr	0.89	0.98	Angew. Chem. Int. Ed. 2023, 62, e202308070
Fe-N-C	0.89	0.97	Angew. Chem. Int. Ed. 2021, 60, 25296
$CuCo_2O_{4-x}S_x$/NC-2	0.88	0.75	Adv. Mater. 2023, 35, 2303488
Fe/Meso-NC-T	0.87	0.97	Adv. Mater. 2023, 34, 2107291
FeH-N-C	0.90	0.97	Adv. Mater. 2023, 35, 2210714
DAF-COF	0.73	0.81	Adv. Mater. 2023, 35, 2209129
Fe1-HNC-500-850	0.84	0.93	Adv. Mater. 2020, 32, 1906905
Ni-N_4/GHSs/Fe-N_4	0.83	0.93	Adv. Mater. 2020, 32, 2003134
PA@Fe-ZIF-8	0.91	0.98	J. Am. Chem. Soc. 2023, 145, 3647
P/Fe–N–C	0.91	0.99	J. Am. Chem. Soc. 2023, 145, 3647
Fe–N/P–C–700	0.86	0.94	J. Am. Chem. Soc. 2020, 142, 2404
Cu-N-C	0.83	0.95	J. Am. Chem. Soc. 2021, 143, 14530
FeCoNC/SL	0.87	0.94	Adv. Sci. 2023, 10, 2205889
Co-N/C-MnO	0.66	0.78	Adv. Funct. Mater. 2022, 2210143
Cu/CNT-8	0.86	0.93	Nat. Commun. 2021, 12, 6335
Co-N-HCS-900	0.87	0.96	Nat. Commun. 2023, 14, 7210

Table S5. Comparison of the performance of solid-state Al-air batteries with various cathode catalysts reported in recent literature.

Catalysts	Electrolyte	Voltage	Discharge time	Reference
**Fe_3C-Fe_1/CNT**	**11.0 M KOH PAA gel**	**1.75 V**	**1.65 V for 28 h @1 mA cm^{-2}**	**This work**
Mn_xO_y/Ag	1:1 Quasi-solid electrolyte	1.62 V	1.65 V for 100 min @1~21 mA cm^{-2}	Adv. Sci. 2023, 10, 2304214
Co_3Fe_7@$Co_{5.47}$N/NCF	18.0 M KOH PAA gel	1.4 V	1.4 V for 12 h @5 mA cm^{-2}	Adv. Sci. 2023, 7, 2000747
W SAs/WNNC-5	11.0 M KOH PAA gel	1.96 V	1.61 V for 4 h @1 mA cm^{-2}	Adv. Sci. 2022, 9, 2105192
P-CD/G Aerogel	18 M KOH PVA gel	1.20 V	1.17 V for 1500 s@2.0 mA cm^{-2}	Adv. Energy Mater. 2020, 10, 1902736
N-NPC-900	6.0 M KOH PANa hydrogel	1.38 V	1.27 V for 30h at 1.0 mA cm^{-2}	Adv. Funct. Mater. 2021, 31, 2103632
FeCo-N-HCN	4.0 M NaOH PVA-PEG polymer gel	1.50 V	1.41 V for 10000 s@50 mA cm^{-2}	Adv. Funct. Mater. 2021, 31, 2011289
FeNi SAs/NC	8.4 M KOH PAA gel	1.74 V	1.65 V for 10 h@1 mA cm^{-2}	Adv. Energy Mater. 2021, 11, 2101242
P-doped carbon dot/graphene aerogel	1.6 M KOH PVA gel	1.2 V	1.17 V for 1500 s@2.0 mA cm^{-2}	Adv. Energy Mater. 2020, 10, 1902736

Table S6. Performance comparison of H_2/O_2 fuel cells using non-precious metal catalysts as the cathode.

Catalysts	Catalyst loading (mg/cm^2)	Open circuit voltage(V)	Peak Power density (mW/cm^2)	Reference
**Fe_3C-Fe_1/CNT**	**4.0**	**0.91**	**0.716**	**This work**
FeN_4-hcC	3.0	0.91	0.592	Adv. Mater. 2023, **35**, 2300907
Fe-NC	4.0	0.94	0.670	Adv. Mater. 2023, **35**, 2302666
Cyan-Fe-N-C	4.0	0.93	0.870	Adv. Mater. 2023, **35**, 2305945
P-Fe-N	3.0	0.96	0.650	Adv. Mater. 2021, **33**, 2006613
FeNC-1100	4.0	0.96	0.687	Adv. Mater. **2023**, 35, 2204474
Fe–NC/CeO_2	4.0	0.92	0.403	Angew. Chem. Int. Ed. 2023, **62**, e202306166
Co_4/Fe_1@NC	4.0	0.93	0.840	Angew. Chem. Int. Ed. 2023, **62**, e202303185
FeN_3OS	3.0	0.97	0.740	Angew. Chem. Int. Ed. 2021, **60**, 25296
Fe-AC-CVD	4.0	0.92	0.601	Nat. Energy. 2022, **7**, 652
Fe-N-C/Ta-TiO_x	6.0	0.95	0.700	Nat. Energy. 2022, **7**, 281
Fe-C-N_{950}	4.8	0.94	0.680	J. Am. Chem. Soc. 2020, **142**, 5477
SA-Fe-NGM	4.0	0.96	0.634	J. Am. Chem. Soc. 2022, **21**, 9280
HP-FeN_4	4.0	0.98	0.700	Energy Environ Sci. 2020, **13**, 111
Fe_2N_6	4.0	0.91	0.845	Matter 2020, **3**, 509
Fe-N/CNT-2	4.0	0.92	0.360	Adv. Funct. Mater. 2019, 29, 1906174
